# The Future of Epigenetics: Emerging Technologies and Clinical Applications

**DOI:** 10.1021/acsptsci.5c00729

**Published:** 2026-02-25

**Authors:** Kavita A. Iyer, Rumiana Koynova-Tenchov, Janet M. Sasso, Trupti Thite, Yi Deng, Qiongqiong Angela Zhou

**Affiliations:** † 41485 CAS, a Division of the American Chemical Society, Columbus, Ohio 43210, United States; ‡ ACS International India Pvt. Ltd., Pune 411044, India

**Keywords:** epigenetics, DNA methylation, histone modification, noncoding RNA, chromatin remodeling, epitranscriptomics

## Abstract

Epigenetics, the study of heritable changes in gene expression that do not involve alterations to the DNA sequence, has emerged as a transformative field in biology and medicine, revealing how gene expression is modulated in response to internal and external cues. Its applications span from understanding fundamental biological processes and disease mechanisms to developing novel diagnostics and therapies, making it a cornerstone of modern biomedical research. This report explores data from the CAS Content Collection to examine recent transformative advances in epigenetic research, highlighting technological innovations that are revolutionizing our understanding of gene regulation and therapeutic applications. Environmental epigenetic research shows strong interest, demonstrating how external factors induce heritable epigenetic changes with implications for transgenerational inheritance. These findings bridge environmental exposures with health outcomes across generations, informing public health strategies and personalized medicine approaches. Disease applications span multiple pathologies, with cancer research leading epigenetic studies. Emerging applications in aging research, metabolic diseases, and autoimmune conditions demonstrate the field’s expanding clinical relevance. Clinical translation has achieved significant success, with 13 FDA-approved epigenetic drugs primarily targeting hematological malignancies through HDAC inhibitors (6 drugs) and DNMT inhibitors (2 drugs). The robust clinical pipeline includes 37 ongoing trials across novel targets, with encouraging diversification beyond oncology into metabolic, neurological, and inflammatory disorders.

## Graphical abstract

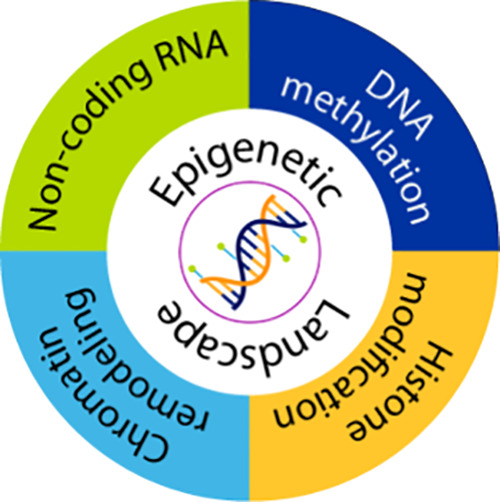

Epigenetics encompasses heritable changes in gene expression that occur without DNA sequence alterations, mediated through reversible modifications to DNA, histones, and RNA, as well as chromatin remodeling.^1–6^ These mechanisms include DNA methylation for gene silencing,^7,8^ histone modifications affecting chromatin accessibility, regulatory noncoding RNAs (ncRNAs),^9,10^ and structural chromatin changes that modulate transcriptional activity.^11–13^

Epigenetic regulation is fundamental to developmental biology,^14,15^ disease pathogenesis (including cancer,^16^ diabetes,^17^ neurodegenerative,^18,19^ and autoimmune disorders),^20–22^ gene-environment interactions,^23^ transgenerational inheritance,^24,25^ and therapeutic development.^26^ By elucidating how genes are dynamically regulated beyond the static genome, epigenetics bridges genetics and environmental influences, offering insights into disease mechanisms and innovative therapeutic strategies.

The commercial significance of epigenetics has expanded dramatically, with the global epigenetics market valued at USD 1.84 billion in 2023 and projected to reach USD 6.77 billion by 2033.^27^ This growth is driven by more than 10 U.S. FDA-approved epigenetic drugs including azacitidine (Vidaza) and vorinostat (Zolinza) for cancer treatment,^28^ and with over 35 epigenetic therapies in clinical trials predominantly targeting various malignancies but starting to show diversification beyond cancer. Major pharmaceutical companies including Merck,^29^ and Celgene^28,30^ have invested significant capital and continue to do so in epigenetic drug development.

In this report, we explore data from the CAS Content Collection,^31^ the largest human-curated collection of published scientific information, to outline the progress made in epigenetic research, to identify key emerging concepts and challenges, and its societal impact, in an effort to understand how epigenetics shapes the future of gene regulation and inheritance. This comprehensive analysis examines the scientific foundations, clinical applications, and technological innovations shaping modern life sciences and healthcare.

The CAS Content Collection contains over 120,000 epigenetic-related publications (2000–2024), demonstrating a steep and continual growth in publications over the last two decades (Figure 1A). The field is dominated by journal articles (97%), with patents comprising only 3% of publications (Figure 1A, inset), indicating that epigenetics remains primarily in the discovery and validation phase, though their sharp increase indicates growing commercial interest and translational potential in this rapidly evolving field. Notably, epigenetic publications have outpaced genetics publications since 2014 (Figure 1B). The emergence of epitranscriptomics as a distinct field^32^ especially noticeable after 2020 (Figure 1B), highlights the expanding scope of epigenetic regulation beyond DNA and histone modifications. This growth trajectory aligns with substantial funding increases, as evidenced by total research funding climbing from USD 200 million in 2004 to over USD 4.5 billion in 2024, supporting approximately 2,700 projects annually^33^ (Figure 1C). The consistent rise in both project numbers and funding levels, with average project funding increasing from USD 1.2 million to USD 1.7 million, underscores sustained governmental and private sector commitment to advancing epigenetic research and its clinical translation.

**Figure 1:**
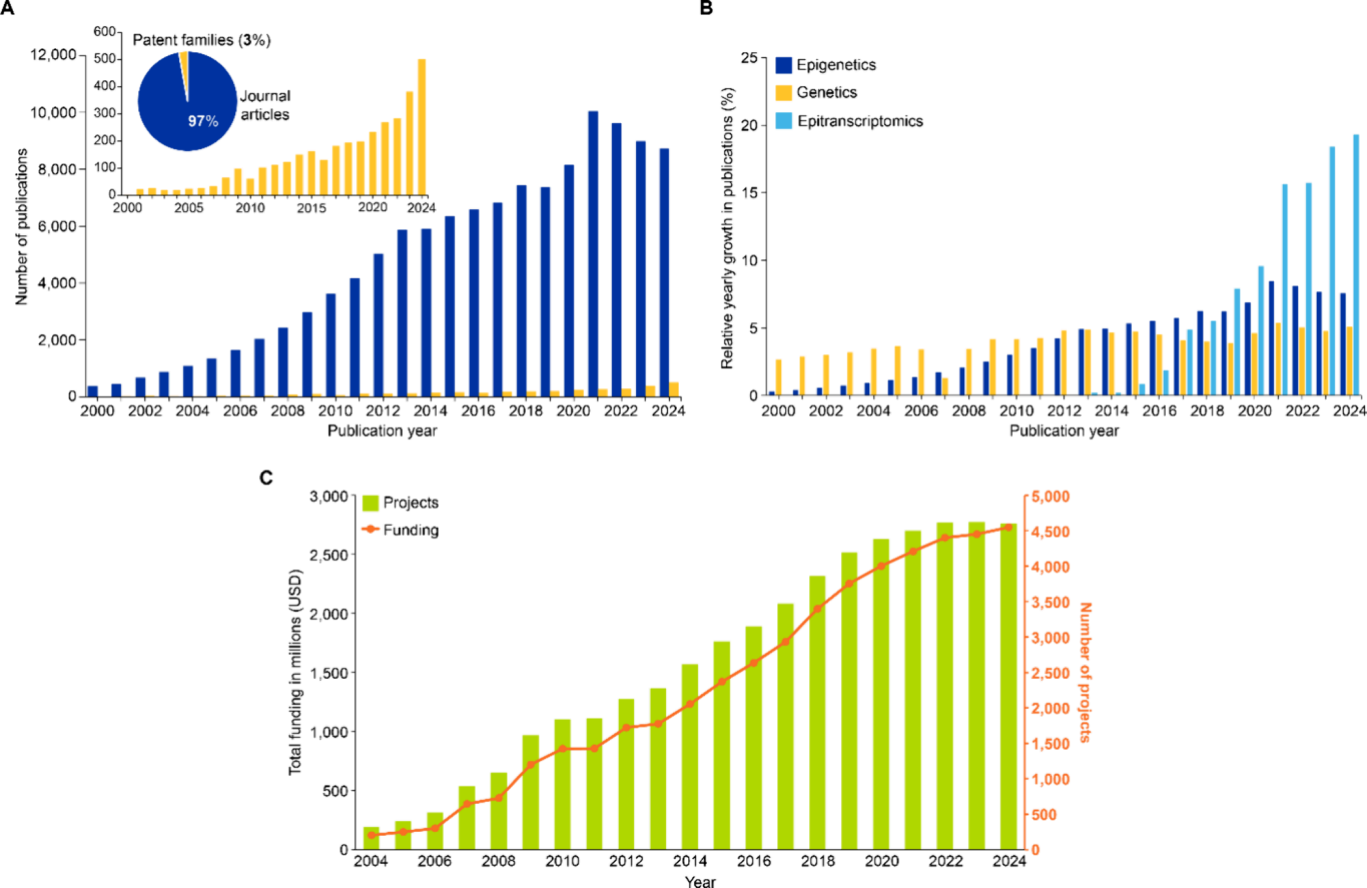
(A) Publication trends for epigenetic-related documents and (B) relative yearly growth in publications related to epigenetics, genetics, and epitranscriptomics. Data includes journal articles and patents extracted from the CAS Content Collection for the period 2000–2024. (C) Trends for number of projects funded and total funding granted to epigenetics related projects by the NIH (https://reporter.nih.gov).^33^

For detailed insights on publication-related analysis please see Figures S1–S4 in Supporting Information. The analysis includes identification of leading research organizations in terms of research output and impact separately (Figure S1), as well as using a combination of both research output and impact (Figure S2), leading commercial and noncommercial patent assignees engaged in filing patents related to epigenetics and their geographical distribution (Figure S3), and leading scientific journals engaged in publishing epigenetic-related research (Figure S4).

## Core Mechanisms of Epigenetic Regulation

Epigenetic mechanisms regulate gene expression through chemical modifications of DNA and chromatin structure without altering the DNA sequence. These mechanisms comprise four main classes: DNA methylation, histone modifications, ncRNA regulation, and chromatin remodeling (Figure 2), forming a layer of control in the cells that regulates gene expression and silencing.^4,6^

**Figure 2:**
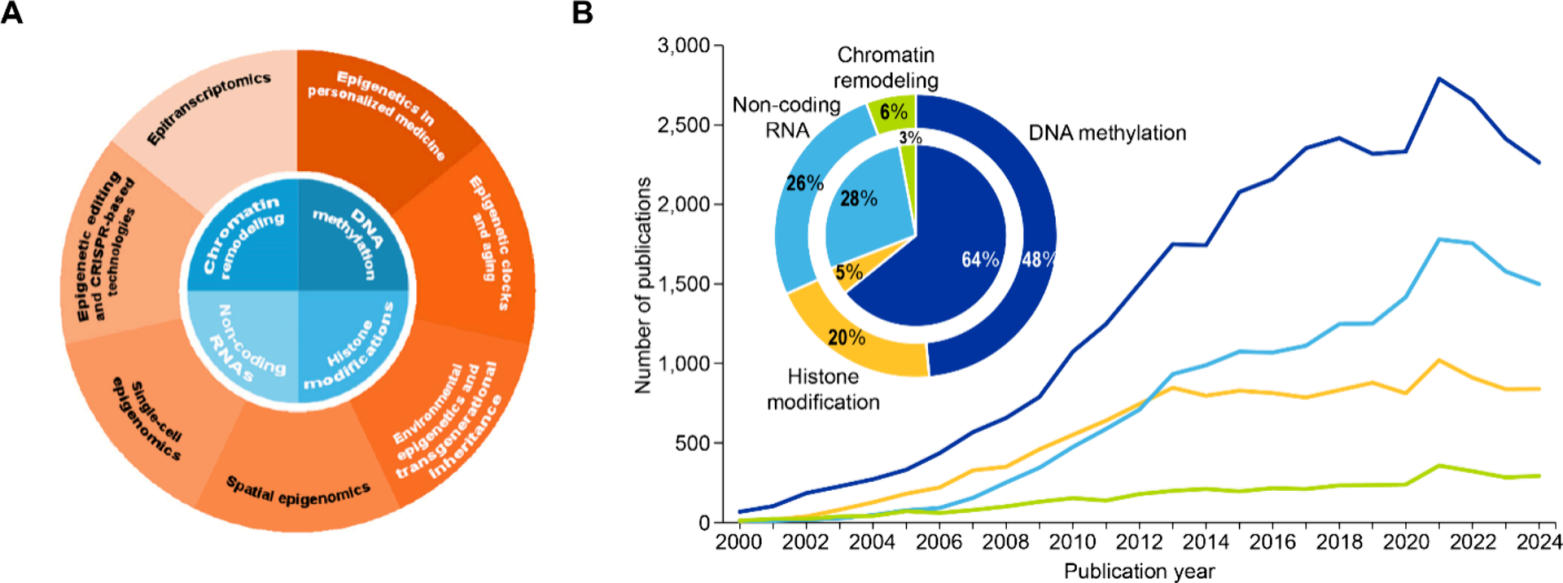
(A) Core mechanisms of epigenetic regulation (inner pie chart) and recent advances (outer donut chart). (B) Publication trends of epigenetic mechanisms based on journal and patent publications from the CAS Content Collection for the period 2000–2024. Outer donut and inner pie chart in the inset image represent journal and patent publications, respectively.

Our analysis of epigenetic-related publications indicates that the early 2000s exhibited limited research output and represents the foundational era when epigenetics was being established as a field. Subsequently, all four mechanisms show accelerated growth, with DNA methylation experiencing the steepest growth, dominating the research landscape, both in terms of journal and patent publications, reflecting its status as the most established and clinically relevant epigenetic modification. This dominance is evident in the sustained growth reaching approximately 2,800 publications annually (Figure 2B). The dominance of DNA methylation research aligns with its clinical applications in cancer diagnostics^34^ and therapeutics,^35^ where methylation biomarkers^36,37^ and DNA methyltransferases (DNMT) inhibitors^38^ have achieved regulatory approval.

ncRNAs and histone modifications also experienced growth, indicating increasing recognition of multiple regulatory layers. The growth in ncRNA research correlates with advances in RNA sequencing and CRISPR-based RNA targeting technologies, enabling functional characterization of previously inaccessible regulatory elements.^39–43^ The emergence of chromatin remodeling as a distinct research focus around 2008 reflects technological advances enabling genome-wide chromatin accessibility studies (Figure 2B).

The sustained interest across all mechanisms, despite recent plateaus, possibly reflective of a shift from discovery-based research to translational applications, suggests continued recognition of epigenetic targets for drug development, particularly in oncology and neurological disorders where epigenetic dysregulation is well-established.

Discussed briefly below are the four major mechanisms of epigenetic modifications.

### DNA Methylation: The Primary Epigenetic Mark

DNA methylation is a fundamental epigenetic mechanism involving addition of methyl groups (−CH_3_) to the cytosine rings at CpG dinucleotides in DNA, primarily resulting in transcriptional repression. This modification plays a critical role in X-chromosome inactivation,^44^ genomic imprinting,^45^ and transposon suppression.^46,47^

DNA methylation is catalyzed by DNA methyltransferases (DNMTs). DNMT1 maintains existing methylation patterns during replication,^48,49^ while DNMT3*a*/3b establishes new methylation patterns during development or in response to environmental cues.^50^ DNMT3L, lacking catalytic activity and mainly expressed in early development, is restricted to the germ cells and thymus in adulthood.^51,52^ DNMTs target CpG islands, regions with high frequency of CpG dinucleotides, often located near gene promoters.^8,53,54^

Aberrant methylation patterns have been identified in various diseases including cancer,^55^ cardiovascular diseases,^56,57^ mental health disorders,^58^ Alzheimer’s disease,^59^ autism,^58,60^ and metabolic syndromes.^61,62^ Hypermethylation of tumor suppressor gene promoters silences their expression promoting oncogenesis,^63^ while global hypomethylation can activate oncogenes and cause genomic instability.^64,65^ DNA methylation patterns change with age,^66^ including the accumulation of errors and loss of fidelity in methylation maintenance.^67,68^

### Histone Modifications: Dynamic Chromatin Regulation

Histone modifications are an epigenetic mechanism, since they can be inherited through cell division and can be influenced by environmental factors, potentially impacting development and disease states.^69^

Post-translational histone modifications such as acetylation, methylation, phosphorylation, and ubiquitination, alter chromatin structure and gene accessibility. Histone acetyltransferases (HATs) and deacetylases (HDACs) regulate acetylation by catalyzing addition and removal of acetyl groups from histones, while methyltransferases (HMTs) and demethylases (HDMs) control methylation. These modifications primarily occur on lysine, arginine, serine, and threonine residues in histone tails, protrusions from the nucleosome core with increased accessibility.^70–73^

Histone modifications affect the structure of chromatin, which is DNA packaged around histone proteins,^74,75^ regulating gene expression, DNA repair, and chromosome condensation during mitosis.^74–76^ Aberrant histone modifications can disrupt gene expression patterns and contribute to tumor development and metastasis,^77^ and are linked to other diseases and disorders such as neurodegenerative^78^ (Alzheimer’s,^79,80^ Huntington’s^81,82^ ), autism,^83^ aging-associated chromatin changes,^84^ and immune dysregulation.^85,86^

Histone modifications often work in combinations, forming a ″histone code″, with combinations of modifications on different residues synergizing or antagonizing to fine-tune chromatin states.^87,88^ Histone modifications are a dynamic and versatile system for regulating chromatin structure and gene expression, and their reversible nature makes them promising targets for therapeutic interventions.^89–91^

### Noncoding RNAs (ncRNAs): Epigenetic Regulators beyond the Genetic Code

ncRNAs constitute a diverse class of regulatory molecules that play critical regulatory roles in gene expression and chromatin dynamics without encoding proteins. These RNAs fine-tune gene processing by targeting mRNA for degradation or modulating transcriptional machinery.^92,93^

While structural RNAs (tRNA, rRNA, small nuclear (snRNA), small nucleolar (snoRNA)) maintain essential cellular functions, three classes have emerged as primary epigenetic regulators: microRNAs (miRNAs), long noncoding RNAs (lncRNAs), and PIWI-interacting RNAs (piRNAs).^10,94^

Our analysis of epigenetic-related publications indicates miRNAs dominated early investigations (2006–2014) appearing to reach a peak in 2021 (Figure 3). This trajectory reflects the field’s maturation from discovery to therapeutic application, with multiple miRNA-based therapies now in clinical trials.^95,96^ lncRNAs^97^ emerged later but demonstrated remarkable growth from 2012 onward with this surge correlating with technological advances in RNA sequencing and functional characterization methods.^98,99^ Small interfering (siRNA) research appears to maintain steady output reflecting its established role in RNA interference therapeutics.^100,101^

**Figure 3:**
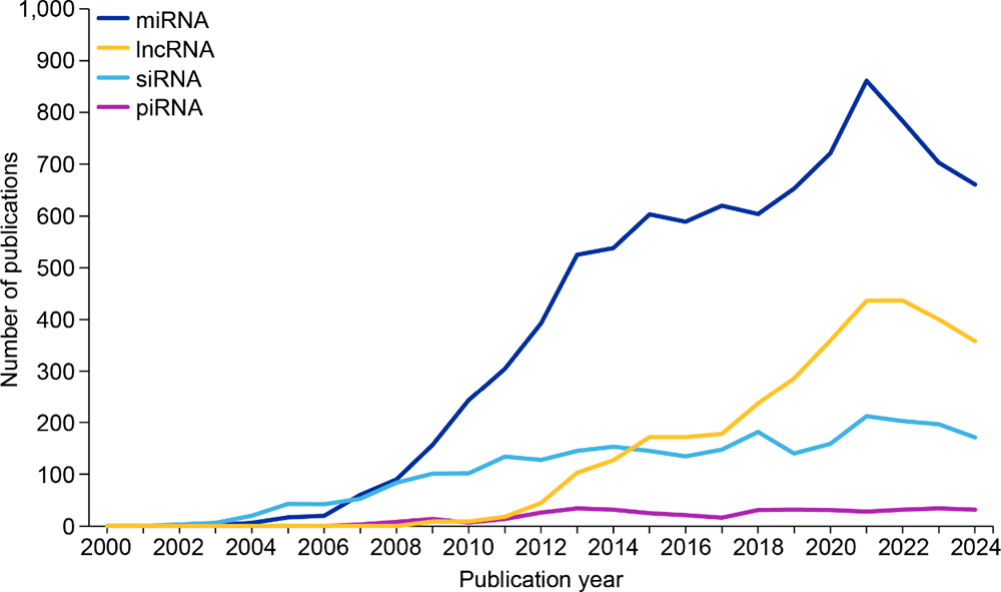
Publication trends of various ncRNAs in the epigenetics data set. Data includes journal articles and patents extracted from the CAS Content Collection for the period 2000–2024.

The modest but consistent publication trajectory of piRNA research (Figure 3) reflects the specialized nature of piRNA biology, which primarily operates in germline cells^102^ and early embryonic development,^103–106^ limiting its study to specific biological contexts compared to the ubiquitously expressed miRNAs and lncRNAs. However, emerging evidence suggests piRNAs function beyond germline contexts.^107^ Somatic piRNA expression has been detected in neurons, where they regulate memory formation and storage^108^ with implications in neurodegenerative diseases.^109,110^ In cancer, aberrant piRNA expression patterns correlate with genomic instability and tumor progression,^111,112^ with specific piRNAs serving as biomarkers for early detection.^113,114^ The stable but modest publication rate for piRNA research likely reflects the field’s technical challenges: the complexity of the ping-pong amplification cycle, the requirement for specialized PIWI proteins, and the predominant germline expression pattern that necessitates specific model systems. As technologies for studying and manipulating piRNAs improve, we may witness an acceleration in publications similar to the lncRNA surge observed after 2012, particularly if somatic functions and therapeutic applications continue to emerge.

The overall publication trends demonstrate ncRNA research’s evolution from mechanistic discovery (2000s) through functional characterization (2010s) to current therapeutic translation (2020s), with each RNA class following distinct developmental trajectories based on biological complexity and clinical applicability.

#### Mechanisms of ncRNA-Mediated Epigenetic Regulation

ncRNAs orchestrate epigenetic regulation through five principal mechanisms:^115–121^

(i) Chromatin remodeling: lncRNAs recruit chromatin-modifying complexes to specific genomic loci, influencing chromatin structure and gene expression. For example, X inactive specific transcript (XIST) mediates X-chromosome inactivation by recruiting polycomb repressive complex 2 (PRC2).
(ii) Transcriptional regulation: miRNAs and lncRNAs modulate transcription by interacting with transcription factors or RNA polymerases. For example, the lncRNA HOX transcript antisense intergenic RNA (HOTAIR) recruits PRC2 to repress genes on a different chromosome.
(iii) Post-transcriptional control: miRNAs bind to the 3′ untranslated regions (UTRs) of target mRNAs (mRNAs), leading to degradation or translational inhibition. lncRNAs can act as molecular sponges to sequester miRNAs, preventing them from targeting mRNAs.
(iv) RNA modification: Some ncRNAs guide RNA methylation (e.g., N6-methyladenosine, m^6^A) or editing, affecting RNA stability and translation.
(v) Genome defense: piRNAs and siRNAs suppress transposable elements, protecting genomic integrity. siRNAs can guide heterochromatin formation in regions with repetitive sequences.

### Chromatin Remodeling: Structural Gene Regulation

Chromatin remodeling dynamically modifies chromatin architecture between euchromatin (open, transcriptionally active) and heterochromatin (condensed, transcriptionally silent) states. The basic unit of chromatin is the nucleosome, composed of DNA wrapped around an octamer of histone proteins (H2A, H2B, H3, and H4).^122^ Chromatin remodeling is carried out by ATP-dependent complexes, such as switch/sucrose nonfermentable (SWI/SNF),^123^ imitation switch (ISWI),^124,125^ chromodomain helicase DNA (CHD)-binding proteins,^126^ and INO80 families,^127^ utilizing ATP hydrolysis to power repositioning, ejection, or restructuring of nucleosomes.^128–130^

Remodeling mechanisms include nucleosome sliding,^131,132^ histone eviction/exchange, and chromatin compaction/decompaction.^129,133^ These processes work in concert with DNA methylation and histone modifications to regulate gene expression, cell differentiation, and identity maintenance.^134–136^

Chromatin remodeling dysregulation is linked to cancer,^137,138^ neurological disorders,^139^ and developmental diseases.^140,141^ For example, mutations in chromatin remodelers like AT-rich interaction domain 1A (ARID1A) (a component of the SWI/SNF complex) are frequently observed in malignancies.^142,143^ Chromatin remodelers represent promising therapeutic targets for reversing aberrant gene expression.^144^ Advances in single-cell technologies are revealing heterogeneity of chromatin states within cell populations, providing deeper insights into cell-specific regulation.^145–147^ Increasingly, chromatin remodeling is being recognized as a mediator of environmental influences (diet, stress, toxins, etc.) on gene expression and health.^148–150^ Future research directions include elucidating remodeler recruitment mechanisms, understanding ncRNA-chromatin interplay, and developing targeted epigenetic therapies.

## Recent Advances in Epigenetic Mechanisms

Recent progress has elucidated how epigenetic modifications including DNA methylation, histone modifications, ncRNA regulation, and chromatin remodeling contribute to disease pathogenesis, particularly in cancer.^151–156^ These insights have driven the development of targeted epigenetic therapies, with emphasis on novel drug combinations that can simultaneously and effectively target multiple epigenetic pathways to enhance treatment efficacy. Emerging research on miRNAs^157^ and other ncRNAs^119,158^ as epigenetic regulators has identified new therapeutic targets, while advances in individual epigenetic profiling^159,160^ are aiding advancements in personalized medicine approaches.^161–163^

Publication trends from the recent past demonstrate the rapidly expanding interest in these advances (Figure 4), with environmental epigenetics seemingly leading research output, followed by epitranscriptomics showing consistent growth. Fields like epigenomic editing,^164,165^ single-cell epigenomics,^166^ and transgenerational epigenetic inheritance^25,167^ appear to be growing at a moderate pace. Finally, newer fields like spatial epigenomics,^168^ while still emerging with limited publications, represent cutting-edge frontiers in the discipline.

**Figure 4:**
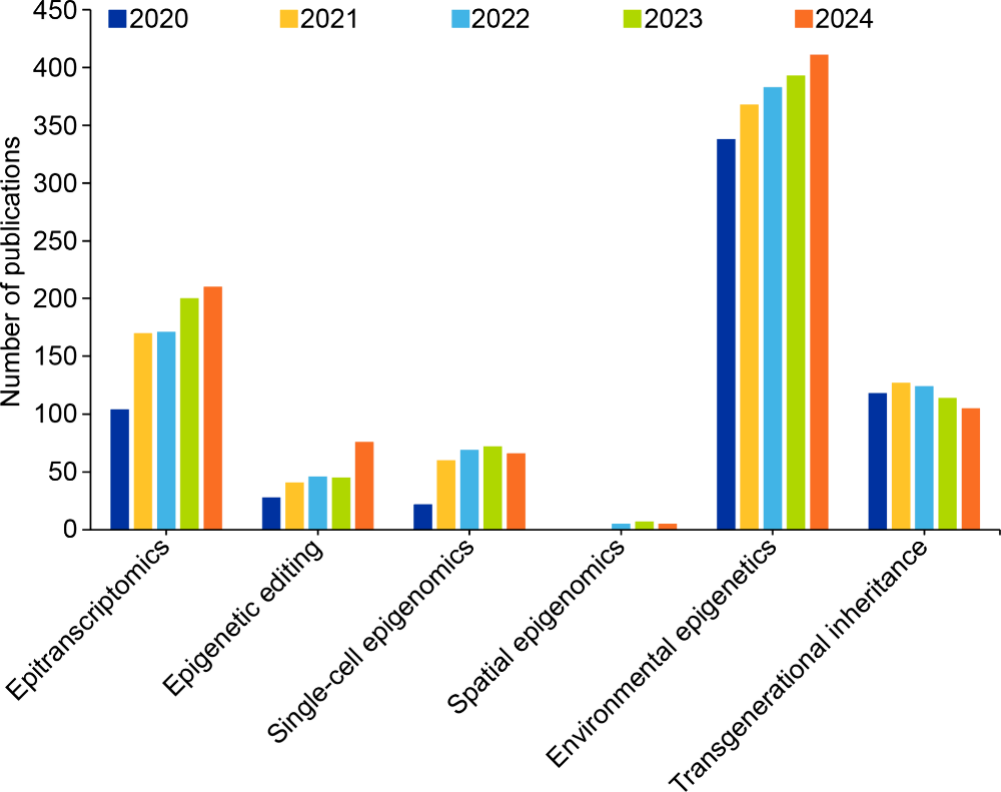
Publication trends for recent advances in epigenetic research. Data includes journal articles and patents extracted from the CAS Content Collection for the period 2000–2024.

### Epitranscriptomics

Epitranscriptomics is an emerging field in epigenetics that focuses on the study of chemical modifications to RNA molecules and their role in regulating gene expression and cellular functions.^32,169^ Just as epigenetics explores modifications to DNA and histones that influence gene activity without altering the underlying DNA sequence, epitranscriptomics investigates how RNA modifications affect RNA stability, translation, splicing, and other processes. This field has gained attention in recent years (Figure 1B and Figure 4) due to its potential to uncover new layers of gene regulation and its implications for health and disease offering new therapeutic and diagnostic opportunities.^170,171^

#### Major RNA Modifications and Regulatory Machinery

Over 170 distinct RNA modifications across mRNA, tRNA, rRNA, and ncRNAs have been identified, with N6-methyladenosine (m^6^A) being the most abundant and extensively studied modification in eukaryotic mRNA.^172–174^ Key modifications include: (i) m^6^A methylation at the N6 position of adenosine, influencing mRNA stability, splicing, export, and translation; (ii) m^5^C methylation at the N5 position of cytosine in mRNA and tRNA, affecting RNA stability and translation; (iii) pseudouridine (Ψ), an isomer of uridine, enhancing RNA stability and translation efficiency; and (iv) A-to-I editing – adenosine-to-inosine deamination, altering base pairing properties and affecting splicing and protein coding.^172–174^

The epitranscriptomic machinery comprises three main protein classes: “writers” are enzymes that add modifications (e.g., METTL3/METTL14 complex for m^6^A methylation), ″erasers″ are enzymes that remove modifications (e.g., fat mass- and obesity-associated protein (FTO) and alkB homologue 5, RNA demethylase (ALKBH5) for m^6^A demethylation), and ″readers″ are proteins that recognize and bind to modified RNA (e.g., YTH domain proteins for m^6^A recognition), which collectively regulate RNA modification dynamics.^172,175–178^

#### Functional Roles and Disease Implications

RNA modifications regulate multiple cellular processes including mRNA stability and decay, translation control, alternative splicing, subcellular RNA localization, and immune recognition of self vs nonself RNA, playing critical roles in embryonic development, stem cell differentiation, and tissue-specific gene expression. Dysregulation of these modifications contributes to various pathologies. In cancer, m^6^A modifications are often altered in tumors^179^ and are linked to tumor progression, metastasis,^180^ and drug resistance.^181^ Aberrant RNA editing and modifications are associated with neurodegenerative diseases including Alzheimer’s,^182,183^ Parkinson’s,^184–186^ and amyotrophic lateral sclerosis (ALS).^187^ Additionally, RNA viruses, including HIV and SARS-Cov-2, exploit host RNA modification machinery to regulate viral replication and immune evasion including epigenetic modifications.^188^

#### Technological Advances and Therapeutic Applications

High-throughput sequencing technologies such as MeRIP-seq (m^6^A specific RNA immunoprecipitation sequencing)^189^ and miCLI (m^6^A individual-nucleotide resolution cross-linking and immunoprecipitation)^189^ enable genome-wide mapping of RNA modifications, complemented by mass spectrometry and chemical labeling techniques for modification detection and quantification.^190,191^ These advances facilitate therapeutic development targeting RNA modification enzymes.

Targeting components of RNA modification machinery (writers, erasers, and readers) is being explored as a strategy for treating diseases. For example, inhibitors of FTO, which are m^6^A demethylases, have shown promise in cancer therapy,^192–194^ while modified nucleotides in mRNA vaccines enhance stability and translation efficiency.^195,196^

#### Future Directions

Current research focuses on deciphering the ″epitranscriptome code,″^197^ developing tools for precise *in vivo* manipulation of RNA modifications, and exploring modifications in ncRNAs.^198^ Single-cell epitranscriptomics is revealing cell-type-specific RNA modification patterns and their functional consequences.^199,200^ Investigations into role of RNA modifications in circadian rhythms,^201,202^ stress responses,^203^ and environmental adaptation^204,205^ are currently being pursued and is expanding our understanding of RNA modification dynamics. As such, the integration of epitranscriptomic and epigenetic mechanisms represents a critical frontier in gene regulation research.

### Epigenetic Editing and CRISPR-Based Technologies

The advent of CRISPR-Cas9 technology has revolutionized genetic engineering, and its application to epigenetics is no exception.^206–208^ Epigenetic editing involves the targeted modification of epigenetic marks without altering the DNA sequence, offering a powerful tool for studying gene regulation and developing novel therapies.

The CRISPR-Cas9 system employs guide RNAs (gRNAs) to direct catalytically inactive Cas9 (dCas9) fused with epigenetic effector domains to specific genomic loci. Key effector domains include DNMT (e.g., DNMT3A), DNA demethylases (e.g., TET1), histone modifiers (e.g., p300, HDACs), and chromatin remodelers, enabling precise addition or removal of epigenetic modifications.^209–211^

CRISPR-Cas9 technology enables selective activation or repression of gene expression by targeting regulatory elements including enhancers and promoters, allowing researchers to directly test the causal roles of specific epigenetic marks in gene regulation,^212,213^ cellular differentiation,^214,215^ and disease.^216^ Additionally, it allows examination of epigenetic inheritance across cell divisions and development, while allowing generation of disease models driven by epigenetic dysregulation such as cancer, neurological, and metabolic disorders.^208,211,217^

Therapeutic applications include silencing disease-causing genes (oncogenes, viral genes) and reactivating beneficial silenced genes (tumor suppressors) in cancer^218^ and hypercholesterolemia^219^ among others.^214,220,221^ This approach offers the potential of highly personalized therapies with minimal off-target effects and applications in cellular reprogramming for regenerative medicine. Key advantages of CRISPR-based epigenome editing include high targeting precision, versatility through interchangeable effector domains, and reduced mutagenic risk compared to gene editing.

Advancing this technology requires developing high-fidelity Cas variants and optimized gRNA designs to minimize off-target effects. Furthermore, multiplexed targeting will enable investigation of complex regulatory networks, while improved delivery methods (viral vectors, nanoparticles) will facilitate clinical translation.^208,217,222^ Large-scale functional screens using CRISPR-based epigenome editing may help identify key epigenetic regulators, and integration with immunotherapy or small-molecule drugs may enhance therapeutic efficacy. Clinical trials are currently evaluating safety and efficacy of these approaches (e.g., NCT06671093 ^223^).^208,217,224^

### Single-Cell Epigenomics

Single-cell epigenomic technologies enable profiling of epigenetic modifications at the resolution of individual cells, providing unprecedented insights into cellular diversity and function,^225–227^ and overcoming drawbacks of traditional bulk sequencing methods which looks at average epigenetic marks across millions of cells. Techniques including single-cell ATAC-seq (assay for transposase-accessible chromatin using sequencing) and single-cell ChIP-seq (chromatin immunoprecipitation sequencing) which profile chromatin accessibility and histone modifications in individual cells and are at the forefront of this trend.^228–234^

Applications span from developmental biology including mapping epigenetic landscapes during embryogenesis,^15,235,236^ cellular and tissue differentiation,^237^ cell fate decisions,^238^ and immune system heterogeneity^239^ to cancer research encompassing uncovering intratumoral epigenetic heterogeneity relevant to clonal evolution,^147,240,241^ drug resistance,^242,243^ and metastasis.^241^ The technology is also used to elucidate epigenetic changes in neurodevelopment,^244^ neurodegeneration,^245^ and immune cell differentiation,^246^ informing understanding of autoimmune diseases and immunotherapy responses. Future directions include integration with spatial transcriptomics,^247^ temporal profiling of epigenetic marks in response to cellular processes or environmental stimuli,^248^ and clinical applications for biomarker identification and discovery as well as development of personalized medicine.^249,250^

### Spatial Epigenomics

Spatial epigenomics is an emerging and cutting-edge technology in epigenetic research that combines the study of epigenetic modifications with spatial context within tissues unlike in single-cell approaches.^251^ This approach bridges single-cell epigenomics and histology, revealing how epigenetic regulation varies across tissue regions and influences cellular organization, function, and communication in health and disease.^251,252^

Applications include mapping tissue patterning during organogenesis guided by epigenetic changes, identifying spatially distinct epigenetic signatures within tumors (core versus invasive margins) which may drive metastasis or drug resistance, characterizing region-specific epigenetic changes in brain tissues or cell types providing insights into neurodevelopment, plasticity, and neurodegenerative diseases, understanding epigenetic regulation across immune cell niches, and influence of epigenetic states in tissue microenvironment.^168,253,254^

### Environmental Epigenetics

Environmental factors such as diet,^255^ stress, toxins, and lifestyle^256^ can induce epigenetic changes affecting gene expression through one of the many epigenetic mechanisms.^257,258^ These modifications contribute to the development of diseases such as cancer,^259^ metabolic disorders,^260^ and neurological conditions^261,262^ by altering cellular function and tissue homeostasis.^263^ Specific influences include: nutrient availability (e.g., folate, vitamin B12) affecting DNA methylation patterns;^264^ chemical exposures (e.g., bisphenol A (BPA), pesticides, air pollutants) altering epigenetic marks leading to long-term health consequences;^265–267^ psychological and physiological stress inducing epigenetic changes in stress-response genes and modulating mental health;^268,269^ and lifestyle factors (e.g., smoking,^270^ alcohol,^271^ physical activity^272^) influencing disease risk via modulating epigenetic states.

### Transgenerational Inheritance

Transgenerational inheritance refers to the transmission of environmentally induced epigenetic changes across multiple generations independent of alterations in the DNA sequence.^273,274^ This phenomenon has been observed in plants^275^ and animals^276^ suggesting that environmental exposures experienced by one generation can affect the health and development of subsequent generations. While reports do exist with regards to humans there are concerns about the extent and significance of it^167,277,278^ with the field plagued by challenges.^279,280^ Mechanisms include germline epigenetic modifications,^281^ epigenetic memory persisting through developmental reprogramming, maternal-fetal crosstalk,^282^ and sperm RNA-mediated information transfer.^283^

Example of transgenerational epigenetic inheritance includes the Dutch Hunger Winter (1944–1945): altered DNA methylation patterns and increased risk of metabolic disorders was observed in subsequent generations of individuals who were subject to famine exposure in Netherlands.^284,285^ Animal studies have also provided examples of transgenerational effects of endocrine disruptors^286,287^ (e.g., vinclozolin^288^) on reproduction, behavior, and disease susceptibility, while in humans paternal smoking^289^ and maternal stress^290^ have been linked to epigenetic changes in offspring.

While the mechanisms underlying transgenerational epigenetic inheritance are not fully understood, their effect on raising the risk profile for diseases such as obesity, diabetes, and cardiovascular disorders in descendants of exposed individuals means that it remains an area of active research. Understanding transgenerational effects can inform policies to reduce exposure to harmful environmental factors, particularly during critical windows of development (e.g., pregnancy).^291–293^ However, challenges persist – difficulty in separating the contributions of genetic and epigenetic factors to inheritance as well as in successfully replicating experiments highlighting the need for rigorous experimental design.

## Epigenetics in Health and Disease

Epigenetic modifications guide embryonic development and cell fate decisions by activating or silencing specific gene sets and epigenetic changes accumulate with age, influencing longevity and age-related diseases.^294,295^ Dysregulation of these processes underlies diverse pathologies including cancer,^16,296^ cardiovascular disorders,^297^ neurodegenerative diseases,^298–300^ and metabolic syndromes.^301,302^ Epigenetic diseases are conditions caused by chemical modifications on DNA and its associated proteins (histones) without altering the underlying DNA sequence. These modifications, called epigenetic marks, can affect gene expression and play a role in various biological processes.^4,303,304^ Summarized in Table 1 are the core epigenetic mechanisms and related biomarkers co-occurring with different diseases as well as information about U.S. FDA approved epigenetic drug availability and ongoing clinical trials.

**Table 1:** Summary of Epigenetic Mechanisms, Biomarkers, U.S. FDA Approved Drugs and Clinical Trials for Various Diseases^a^

	Epigenetic mechanisms				U.S. FDA approved drugs	Clinical trials		Biomarkers			
	DNA methylation	Histone modification	ncRNA	Chromatin remodeling	**√**/X	**√**/X	Highest phase of development	DNA methylation	Histone modification	ncRNA	Chromatin remodeling
Cancer	11,917	4,287	7,506	1,364	**√**	**√**	Phase III	2,202	422	1,530	134
Aging	2,545	793	945	220	X	X	-	479	52	158	19
Neurological	2,147	781	942	194	X	X	-	303	57	198	13
Blood disorders	1,790	759	913	265	X	X	-	182	41	119	20
Cardiovascular	905	391	712	84	X	X	-	143	31	141	6
Metabolic	2,142	722	1,080	111	X	X	-	279	40	160	10
Respiratory	544	202	299	30	X	X	-	74	19	54	4
Autoimmune	927	379	637	52	X	X	-	140	45	149	3
Gastrointestinal	154	59	107	9	X	X	-	30	10	25	2
Infectious	2,281	931	1,454	280	X	**√**	Phase I	278	7	166	13

a The numbers correspond to journal and patent publications from the CAS Content Collection.

### Cancer: Epigenetic Dysregulation in Tumorigenesis

While genetic mutations have long been recognized as drivers of cancer, epigenetic alterations are now understood to play an equally critical role in tumorigenesis.^305^ The epigenetic disease research landscape (Figure 5, pie chart) reveals **cancer**’s dominance with 47% of publications, reflecting both the field’s maturity and clinical translation success with temporal analysis demonstrating cancer research’s sustained growth (Figure 5, inset). Solid tumors account for an overwhelming majority of publications with breast, colorectal, liver, and lung cancer leading in terms of research activity (Figure 5, sankey). Among hematological malignancies, acute myeloid leukemia (AML) dominates, followed by chronic lymphocytic leukemia (CLL) and multiple myeloma (MM). This distribution observed based on publication data from the CAS Content Collection aligns with U.S. FDA-approved epigenetic therapies targeting these malignancies, including azacitidine^306,307^ and decitabine^308,309^ used for treatment of AML and vorinostat for cutaneous T-cell lymphoma.^310^

**Figure 5:**
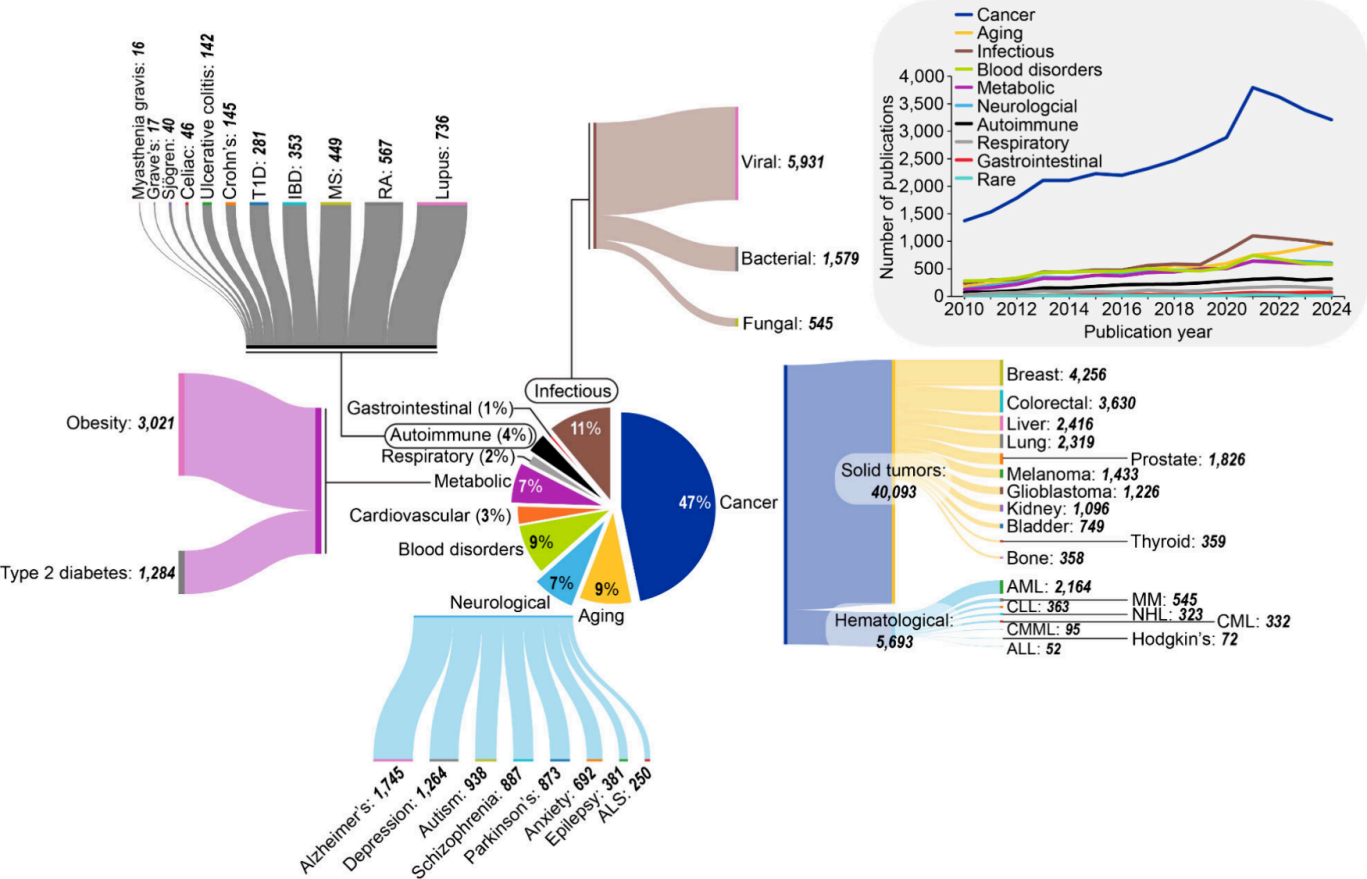
Relative distribution of epigenetic-related publications among various disease types depicted in the pie chart at the center. The individual Sankey charts show the distribution of publications among subtypes of diseases. Inset line graph at the top right shows publication trends of the broader disease categories. Data includes journal articles and patents extracted from the CAS Content Collection for the period 2000–2024.

Epigenetic regulation in cancer involves three primary mechanisms: (i) DNA methylation^311^ – hypermethylation of promoter regions often leads to the silencing of tumor suppressor genes (e.g., p16,^312,313^ BRCA1 ^314,315^ ); global hypomethylation, on the other hand, can activate oncogenes and promote genomic instability;^64^

(ii) histone modifications^77^ – alterations in histone acetylation, methylation, and phosphorylation can change chromatin structure and gene expression – for example, loss of histone acetylation is associated with the repression of tumor suppressor genes, while gain of repressive histone marks (e.g., H3K27me3 ^316,317^ ) can silence critical regulatory genes;
(iii) ncRNAs^318^ – dysregulation of miRNAs (e.g., miR-21 overexpression) can promote cancer progression by targeting tumor suppressors or oncogenes.^16,319,320^

The gene-epigenetic mechanism co-occurrence heatmap (Figure 6A, inset) reveals distinct patterns: DNA methylation’s dominance reflecting its role as an important epigenetic mechanism; chromatin remodeling shows minimal co-occurrence across most genes, suggesting either its role in broader architectural changes rather than gene-specific regulation; while publications associated with ncRNA and histone modifications lie between the two extremes – DNA methylation and chromatin remodeling. Our analysis of gene and disease co-occurrences (Figure 6B) illustrates cancer’s central position, with tumor suppressor genes showing highest connectivity. Other genes with high co-occurrences across multiple cancers include CDKN2A,^321,322^ followed by APC^323,324^ and c-*K*_i_-ras (KRAS^325^). This pattern reflects these genes’ roles as epigenetic hubs subject to methylation-mediated silencing across diverse malignancies.

**Figure 6:**
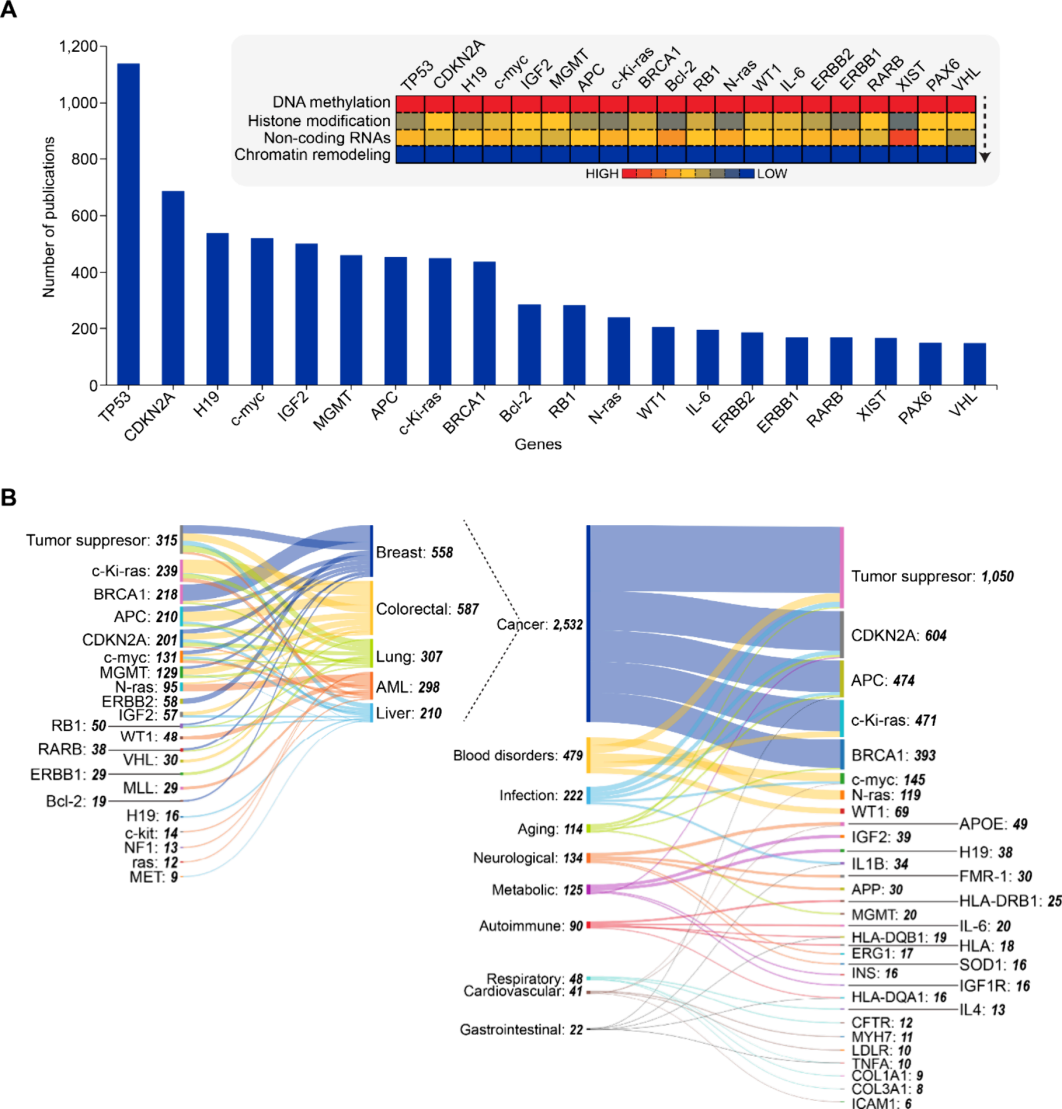
(A) Leading genes associated with epigenetic publications based on CAS indexing. Inset heat map table shows co-occurrence of epigenetic mechanisms with individual genes. (B) Sankey graphs depicting disease and gene co-occurrences in the epigenetics data set (restricted to top 5 genes for each disease indication). Data includes journal and patent publications from the CAS Content Collection for the period 2000–2024.

### Epigenetic Clocks: Quantifying Biological Aging

Epigenetic clocks are a groundbreaking concept in epigenetics implicating computational models that predict biological age based on DNA methylation patterns,^326,327^ emerging as powerful tools for studying aging,^328^ longevity, and age-related diseases.^329^ They can reveal discrepancies between an individual’s biological age and chronological age, providing insights into the aging process and the impact of lifestyle and environmental factors. These models exploit predictable age-related changes in DNA methylation, where specific genomic regions become hypermethylated or hypomethylated in reproducible patterns.^326,330,331^

Research publication trends (Figure 5) demonstrate that aging represents 9% of epigenetic research explicitly mentioning disease and aging, significantly smaller than cancer research (47%), yet showing consistent growth from 2010 to 2024 (Figure 5, inset). This proportion reflects aging research’s specialized but expanding role within the broader epigenetic landscape dominated by cancer applications.

The first-generation epigenetic clock, Horvath’s clock, was developed by Steve Horvath in 2013 and utilizes DNA methylation data from 353 CpG sites across multiple tissues and cell types to estimate biological age,^332^ while Hannum’s clock, developed in the same year,^333^ employs 71 CpG sites, primarily in blood. More recent clocks like GrimAge^334^ and PhenoAge^335^ incorporate additional biomarkers (e.g., plasma proteins) to improve accuracy^336^ and predict mortality and disease risk.^337^

Epigenetic clocks use machine learning algorithms to analyze DNA methylation data and help in prediction of biological age including age acceleration (if epigenetic age > chronological age) or deceleration (if epigenetic age < chronological age), with acceleration associated with increased age-related disease and mortality risk.^327,338^

Applications span intervention studies evaluating antiaging treatments (caloric restriction, exercise, pharmacological agents),^295,339,340^ longevity research enabling researchers to study the effects of genetics, lifestyle, and environmental factors on aging, and age-related disease prediction. In longevity research centenarians and individuals with exceptional longevity often exhibit slower epigenetic aging, providing clues to the genetic and environmental factors that promote healthy aging.^341–343^ Current research explores reversibility through senolytics and epigenetic regulator-targeting compounds.^339^

### Neurological Disorders: Epigenetic Mechanisms in Neurodegeneration

Epigenetic modifications have been linked to neurodegenerative diseases such as Alzheimer’s,^262,344^ Parkinson’s,^345^ and ALS,^346–348^ as well as mental health disorders such as depression,^349^ and schizophrenia.^350^ The substantial research output reflects growing recognition of epigenetic mechanisms in neuropsychiatric and neurodegenerative pathologies (Figure 5). Epigenetic alterations affect genes involved in amyloid-beta production (APP;^182^ Figure 6) and tau phosphorylation in Alzheimer’s disease, with dysregulation of miRNAs,^351^ such as miR-29 ^352,353^ and miR-34 ^354^ being associated with cognitive decline. Parkinson’s disease shows epigenetic changes in mitochondrial function genes (e.g., PINK1 ^355^) and α-synuclein aggregation pathways.^356^ Histone acetylation^78,357^ and DNA methylation^358^ patterns are disrupted in Parkinson’s disease models, affecting neuronal survival.

Autism spectrum disorders^359^ exhibit dysregulation of synaptic genes (e.g., SHANK3 ^360^) through aberrant DNA methylation^361,362^ and histone modifications,^83,363,364^ with environmental factors such as prenatal exposure to valproic acid shown to induce epigenetic changes in animal models.^365^ In epilepsy, epigenetic mechanisms regulate ion channel genes and synaptic plasticity genes, contributing to seizure susceptibility, with histone modifications^366^ and miRNA dysregulation^367^ being observed in epilepsy models. Neurodevelopmental disorders exemplify critical epigenetic regulation as exhibited by mutations in the MECP2 gene, encoding a methyl-CpG-binding protein, causing Rett syndrome^368^ and FMR1 hypermethylation in Fragile X syndrome.^369,370^

Epigenetic alterations are increasingly recognized as biomarkers for neurological disorders.^371^ Methylation patterns of genes such as BDNF^372,373^ and COMT^374^ are associated with cognitive function and psychiatric disorders, while specific histone marks (e.g., H3K27me3) are linked to gene regulatory networks in neurodegeneration.^78^ Circulating miRNAs,^375^ particularly miR-132 ^376,377^ and miR-124 ^378^ have shown potential as noninvasive biomarkers for diagnosis of Alzheimer’s and Parkinson’s disease, respectively, offering potential for early detection and therapeutic monitoring.

### Cardiovascular: Environmental-Epigenetic Interactions

Epigenetic mechanisms contribute to the pathogenesis of various cardiovascular diseases^297,379–381^ though our analysis of publication data suggests smaller fraction of research interest when compared to cancer (Figure 5 and Figure 6). For instance, in atherosclerosis, DNA methylation^57^ and histone modifications^382^ regulate genes involved in inflammation, lipid metabolism, and endothelial function;^383^ hypomethylation of pro-inflammatory genes (e.g., IL-6 ^384^) and hypermethylation of anti-inflammatory genes (e.g., PPARγ^385^) promote plaque formation. Epigenetic changes in genes regulating vascular tone (e.g., ACE,^386^ eNOS^387^) and sodium homeostasis contribute to hypertension; environmental factors such as high-salt diet^388–390^ and stress can induce these epigenetic alterations. Epigenetic regulation of ion channel genes (e.g., SCN5A,^391^ KCNQ1 ^392^) can predispose individuals to arrhythmias. Similarly, epigenetic modifications drive pathological cardiac remodeling, including hypertrophy and fibrosis;^381^ DNA methylation,^393^ histone modifications^394^ and miRNAs (e.g., miR-208 ^395^) are key regulators of these processes. In ischemic heart disease and stroke, DNA methylation^396^ and histone modifications^397^ alter the expression of genes that govern cell survival, stress responses, and inflammatory pathways.

Epigenetic alterations are increasingly documented as biomarkers for cardiovascular disease diagnosis, prognosis, and risk stratification.^398,399^ Methylation patterns of genes such as F2RL3 ^400^ and AHRR^401^ are associated with cardiovascular risk and outcomes. Specific histone marks (e.g., H3K27ac^402,403^ ) are linked to gene regulatory networks in heart failure and atherosclerosis. Circulating miRNAs (e.g., miR-126,^404^ miR-499 ^405^) are used as biomarkers for acute myocardial infarction and heart failure.^398,399,406^

### Metabolic Disorders

Epigenetic modifications regulate genes controlling insulin sensitivity, fat storage, and inflammation in metabolic diseases.^17,407,408^ Environmental factors including diet and exercise induce persistent epigenetic changes contributing to insulin resistance and metabolic dysfunction.^409^ Metabolic diseases represent a sizable fraction of epigenetic research (Figure 5), with obesity studies and type 2 diabetes dominating this field. The substantial publication volume reflects the global epidemic of metabolic diseases^410^ and growing understanding of epigenetic contributions to metabolic dysfunction. The steady but modest growth in metabolic epigenetic research (Figure 5, inset) contrasts with the exponential increase in cancer studies, suggesting significant untapped potential in this area. Recent studies reveal epigenetic transgenerational inheritance of metabolic phenotypes^292,293,411^ with these findings have profound implications for public health interventions targeting metabolic disease prevention across generations.

- Obesity: DNA methylation and histone modifications regulating adipogenesis, appetite control, and energy expenditure genes, with dysregulated miRNAs (e.g., miR-103, miR-143) being associated with obesity and adipose tissue dysfunction.^412^
- Type 2 diabetes (T2D): Epigenetic changes in pancreatic β cells, liver, and skeletal muscle affecting insulin production and sensitivity, including DNA methylation of PPARGC1A and IRS1 genes.^17,413^

- Nonalcoholic fatty liver disease (NAFLD): DNA methylation and histone modifications regulate hepatic lipid metabolism and inflammation, with miRNAs (e.g., miR-34a, miR-122) contributing to disease progression.^414,415^

- Metabolic syndrome: Epigenetic changes in glucose metabolism, lipid metabolism, and blood pressure regulation genes. Metabolic syndrome models characterized by histone modifications and miRNA dysregulation.^11,416,417^

- Cardiometabolic diseases: Epigenetic mechanisms link metabolic dysregulation to cardiovascular complications through DNA methylation of genes including FTO^418^ and ABCA1,^419^ associated with atherosclerosis and hypertension risk.^11,297^ These findings demonstrate epigenetic mechanisms as critical links between environmental exposures and metabolic disease development.

Epigenetic biomarkers for metabolic diseases include: methylation patterns of TXNIP^420,421^ and SREBF1 genes associated with T2D and NAFLD;^422,423^ specific histone marks (e.g., H3K9Ac^424^) linked to metabolic gene regulatory networks; and circulating miRNAs (e.g., miR-375,^425^ miR-21 ^426^) serving as biomarkers for T2D and obesity. These biomarkers^427^ enable early detection, risk stratification, and therapeutic monitoring in metabolic disorders.

### Autoimmune Diseases: Immune Dysregulation through Epigenetic Alterations

Aberrant methylation of immune tolerance genes can activate self-reactive T cells, leading to autoimmune conditions. Despite their clinical importance, autoimmune diseases represent a modest portion of epigenetic research (Figure 5), with steady but limited growth from 2010 to 2024 compared to the exponential increase in cancer studies (Figure 5, inset). This research gap suggests significant potential for expanded investigation in autoimmune epigenetics. Within autoimmune diseases, systemic lupus erythematosus (SLE), rheumatoid arthritis (RA), multiple sclerosis (MS), inflammatory bowel disease (IBD), and type 1 diabetes (T1D)^428^ have emerged as primary research areas.

- Systemic lupus erythematosus (SLE) pathogenesis associated with dysregulated miRNAs (miR-21, miR-148a).^429^
- Rheumatoid arthritis (RA) involves DNA methylation changes in synovial fibroblasts and immune cells promoting joint inflammation, while histone modifications (H3K27ac) regulate pro-inflammatory cytokine production (TNF-α, IL-6).^430^
- Multiple sclerosis (MS) exhibits epigenetic changes in T and B cells affecting immune regulation and myelin destruction genes, with miRNA dysregulation (e.g., miR-326,^431^ miR-155 ^432^) linked to disease progression.
- Type 1 diabetes (T1D) involves DNA methylation and histone modifications in pancreatic β cells and immune cells contributing to autoimmune destruction of insulin-producing cells, with miRNAs (e.g., miR-21, miR-34a) implicated in pathogenesis.^433^
- Inflammatory bowel disease (IBD) such as Crohn’s and ulcerative colitis exhibit epigenetic changes in intestinal epithelial and immune cells driving chronic inflammation along with association with miRNA dysregulation (e.g., miR-21, miR-155).^434–436^

### Epigenetics in Personalized Medicine

Integration of epigenetic data enables personalized treatment strategies based on individual epigenetic profiles, aiding development of highly precise diagnostic tools, prognostic markers, and therapeutic approaches particularly for complex diseases including cancer, neurological disorders, and metabolic conditions.^437,438^ Epigenetic modifications can serve as tissue-specific biomarkers^439^ providing insights into disease mechanisms and progression.

Examples of disease-specific epigenetic patterns include: cancer (tumor suppressor gene hypermethylation and global hypomethylation), neurological disorders (aberrant methylation and histone modifications in Alzheimer’s, Parkinson’s, and autism spectrum disorders), and metabolic diseases (epigenetic changes in glucose and lipid metabolism genes contributing to diabetes and obesity).

Epigenetic biomarkers enable early disease detection and diagnosis, for example through liquid biopsies utilized for detecting cancer-specific DNA methylation patterns in blood or other bodily fluids for noninvasive diagnosis and monitoring,^440^ and prenatal testing using epigenetic markers in maternal blood to assess fetal health.^441,442^ Prognostically epigenetic markers can predict disease outcomes and response to therapy. For example, methylation status predicts outcomes and metastasis likelihood in some cancers,^443,444^ while epigenetic changes in psychiatric disorders may predict medication response.^445^

Epigenetic drugs targeting DNA methyltransferases (DNMTs) and histone deacetylases (HDACs) are clinically established and continue to be developed. DNMT inhibitors (e.g., azacitidine, decitabine) treat myelodysplastic syndromes and AML, while HDAC inhibitors address certain lymphomas and multiple myeloma.^26,446^ Combination therapies integrating epigenetic drugs with chemotherapy and immunotherapy enhance treatment efficacy, representing a key advancement in personalized therapeutic approaches.^26,446^

### Epigenetic Therapeutics: From Mechanisms to Clinical Applications of Epi-Drugs

Epigenetic drugs (epi-drugs) reverse aberrant epigenetic modifications to restore normal gene expression in diseases characterized by epigenetic dysregulation.^26,305,447^ The therapeutic landscape (Figure 7) reveals HDAC inhibitors dominating with 59% of publications, followed by DNMT inhibitors (18%), and RNA modulators (8% for ncRNA and general RNA modulators). Table 2 summarizes mechanisms of action of epi-drugs, with these mechanisms highlighting how epi-drugs target specific components of the epigenetic machinery to restore normal gene expression patterns, making them promising tools for treating cancers and other diseases driven by epigenetic dysregulation. Table S2 lists exemplary epi-drugs approved for clinical application.

- **Histone deacetylase (HDAC) inhibitors** function as broad-spectrum lysine deacylases that target both histone and nonhistone substrates while removing diverse acyl modifications beyond acetylation.^448,449^ HDACs were originally known for preventing the removal of acetyl groups from histones, which relaxes chromatin and increases gene expression, including tumor suppressor genes. However, researchers now recognize that HDACs have much broader substrate specificity.^450,451^ These enzymes deacylate numerous nonhistone proteins including p53, NF-κB, HSP90, α-tubulin, and STAT3, thereby regulating protein stability, subcellular localization, and protein–protein interactions critical for cellular homeostasis.^452^ Beyond acetylation, HDACs remove various acyl modifications including propionylation, butyrylation, crotonylation, and succinylation from lysine residues, expanding their regulatory impact on cellular metabolism and gene expression.^453^ This multifaceted biology explains the pleiotropic effects of HDAC inhibitors in cancer therapy, including induction of apoptosis, cell cycle arrest, and immune modulation. HDAC inhibitors also affect nonhistone proteins, such as transcription factors^454^ and chaperone proteins,^455^ further contributing to their anticancer effects. The dramatic surge in publications from early 2000s to early 2010s followed by stabilization (Figure 7A) indicates transition from discovery to clinical implementation. HDAC inhibitors currently approved for clinical use include vorinostat (Zolinza), Romidepsin (Istodax), belinostat (Beleodaq), panobinostat (Farydak) (canceled by U.S. FDA in 2022 but still approved by European Medicines Agency (EMA)), givinostat (Duvyzat) and chidamide (Tucidinostat) (approved by National Medical Products Administration (NMPA) in China and Pharmaceuticals and Medical Devices Agency (PMDA) in Japan) (Table S2).
- **DNA methyltransferases (DNMT) inhibitors**, representing the second-largest drug class in terms of associated research publications (Figure 7A, inset pie chart), block DNA methyltransferases (DNMTs) aiding reactivation of silenced tumor suppressor genes in cancer.^456–458^ DNMT inhibitors currently approved for clinical use are azacitidine (Vidaza) and decitabine (Dacogen) (Table S2), nucleoside analogs that incorporate into DNA during replication, irreversibly bind DNMTs and preventing methylation. This leads to hypomethylation of DNA and reactivation of silenced tumor suppressor genes and other genes involved in differentiation and apoptosis. Temporal analysis shows steady growth in DNMT inhibitor research reflecting continued optimization of these first-generation epigenetic drugs (Figure 7A).
- **ncRNA modulators** target miRNAs or lncRNAs to influence gene regulation. RNA modulators appear to be a fast-growing category, especially evident after 2018 (Figure 7A), reflecting recognition of miRNAs and lncRNAs as druggable targets for precision medicine.^116,318,483,484^

- **Bromodomain and extra-terminal domain (BET) inhibitors** block the binding of BET proteins to acetylated histones, disrupting the transcriptional activation of oncogenes. BET proteins (e.g., BRD2, BRD3, BRD4) are involved in recognition and binding of acetylated lysines on histones and acting as ″readers″ of epigenetic marks.^459–462^ BET inhibitors (OTX015, CPI-0610, JQ1, I-BET762 etc.; Table S2) remain in clinical trials, with consistent publication growth since 2012 indicating sustained development efforts (Figure 7A).
- **Histone lysine demethylases (KDMs) inhibitors** work by inhibiting enzymes belonging to lysine demethylases (LSDs) or JmjC family *N*-methyl lysine demethylases (JmjC) family of enzymes.^467^ No approved KDM inhibitors so far, however, a KDM4 inhibitor is currently in clinical trial (zavondemstat, NCT05076552 ^485^) with ongoing research directed toward developing more KDM4 inhibitors.^486^
- **Histone methyltransferase (HMT) inhibitors** target enzymes that add methyl groups to specific lysine or arginine residues on histone proteins, which can either activate or repress gene expression depending on the specific histone and the location of the methylation. Inhibiting these enzymes can alter gene expression profiles in cancer cells.^468–470^

- **Protein arginine methyltransferase (PRMT) inhibitors** target protein methyltransferases, enzymes responsible for adding methyl groups to proteins and impacting gene expression and cellular processes.^487–489^ HMT inhibitors (2%) and protein methyltransferase inhibitors such as PRMT and PKMT inhibitors (1% each) remain in early development, while KDM inhibitors (3%) show emerging therapeutic potential (Figure 7A, inset pie chart).
- **Isocitrate dehydrogenase (IDH) inhibitors** target mutant forms of IDH enzymes, such as IDH1 and IDH2, that produce the oncometabolite 2-hydroxyglutarate, causing DNA hypermethylation and block cellular differentiation by accumulating in cells and inhibiting enzymes involved in epigenetic regulation, such as TET proteins and histone demethylases.^479–482^ Mutant IDH enzymes are common in certain cancers such as AML, gliomas, etc.^490^ and inhibition of mutant IDHs can restore normal cellular differentiation.^491,492^ Ivosidenib (Tibsovo) and enasidenib (Idhifa) are examples of IDH inhibitors approved for clinical use (Table S2).
- **Enhancer of zeste homologue 2 (EZH2) inhibitors** function as specific HMT inhibitors, reducing H3K27me3 levels and reactivating silenced tumor suppressor genes which can restore normal cellular differentiation and inhibit tumor growth, particularly in cancers with EZH2 mutations or overexpression. EZH2 is the catalytic subunit of the polycomb repressive complex 2 (PRC2) and mediates the methylation of histone H3 at lysine 27 (H3K27me3), a mark associated with gene silencing.^476–478^ In cancer, EZH2 is often overactive, leading to the silencing of tumor suppressor genes. Approved EZH2 inhibitors are tazemetostat (Tazverik) and valemetostat tosilate (Ezharmia) (only approved by PMDA in Japan) (Table S2).
- **Dual-action or multiepigenetic modulators** combine mechanisms targeting multiple epigenetic pathways. These drugs may enhance therapeutic efficacy in complex diseases.^493–495^

- Combination strategies integrating epi-drugs with chemotherapy, immunotherapy, and targeted agents show promise for enhanced efficacy.^496^

**Figure 7:**
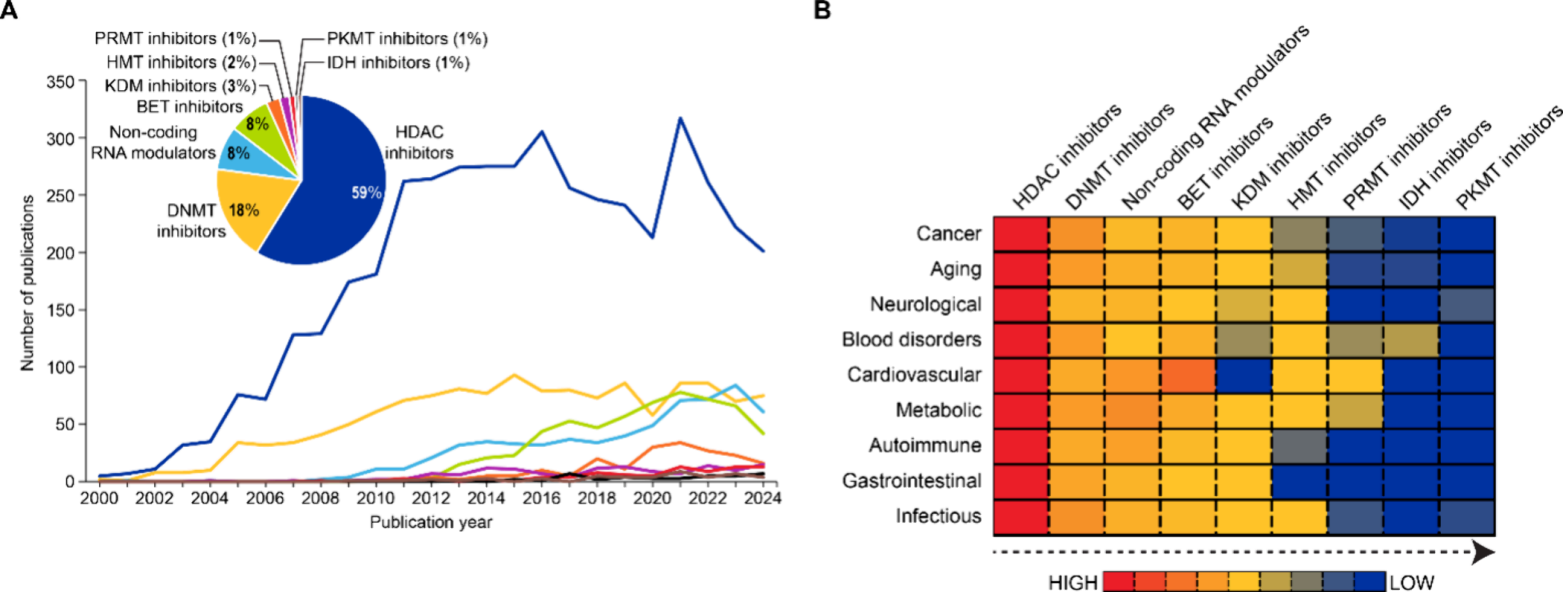
(A) Distribution and publication trends of epi-drug classes in the epigenetics data set. (B) Heat map showing co-occurrence of epi-drug classes with diseases. Data includes journal and patent publications from the CAS Content Collection for the period 2000–2024. Abbreviations used: HDAC, histone deacetylase; DNMT, DNA methyltransferase; BET, bromodomain and extra-terminal domain; KDM, histone lysine demethylase; HMT, histone methyltransferase; PRMT, arginine methyltransferase; PKMT, lysine methyltransferase; IDH, isocitrate dehydrogenase.

**Table 2:** Summary of Mechanisms of Action of Known Classes of Epi-Drugs

Epi-drug class	Target	Mechanism	Outcome
HDAC inhibitors^448–453^	Histone deacetylases (HDACs)	Inhibit removal of acetyl and other acyl groups (propionyl, butyryl, crotonyl, succinyl) from histone and nonhistone proteins (p53, NF-κB, HSP90, α-tubulin, STAT3)	Chromatin relaxation, reactivation of silenced genes, modulation of protein stability/localization, induction of apoptosis, cell cycle arrest, immune activation
DNMT inhibitors^456–458^	DNA methyltransferases (DNMTs)	Inhibit DNA methylation, leading to hypomethylation	Reactivation of silenced tumor suppressor genes
BET inhibitors^459–462^	BET proteins (BRD2, BRD3, BRD4)	Block binding to acetylated histones, disrupting transcription	Downregulation of oncogenes (e.g., MYC)
KDM inhibitors^463–467^	Histone lysine demethylases (KDMs)	Prevents histone demethylation	Downregulation of oncogenes
HMT inhibitors^468–470^	EZH2 (e.g., PRC2 complex)	Inhibit histone methylation (e.g., H3K27me3)	Reactivation of silenced tumor suppressor genes
PRMT inhibitors^471,472^	Protein arginine methyltransferases (PRMTs)	Decreases histone methylation	Helps overcome drug resistance (e.g., by regulating the Wnt/β-catenin signaling pathway)
PKMT inhibitors^473–475^	Protein lysine methyltransferases (PKMTs)	Decreases histone methylation	Helps overcome drug resistance
EZH2 inhibitors^476–478^	EZH2 (PRC2 complex)	Inhibit H3K27 methylation	Reactivation of silenced genes, restoration of differentiation
IDH inhibitors^479–482^	Mutant IDH1/IDH2 enzymes	Reduce 2-HG levels, restoring normal epigenetic regulation	Restoration of cellular differentiation, inhibition of tumor growth

Co-occurrence analysis of diseases with specific epi-drugs shown as a heat map in Figure 7B, reveals disease-specific research patterns across drug classes. For all of the broad disease categories considered, HDAC inhibitors show the highest co-occurrence reflecting their broad therapeutic potential. Similar patterns were observed for DNMT inhibitors and ncRNA modulators. Cardiovascular and metabolic diseases demonstrate moderate research activity, primarily focusing on HDAC and DNMT inhibitors for reversing pathological cardiac remodeling and metabolic dysfunction, though BET inhibitors had a slightly higher co-occurrence while KDM inhibitors had little to no co-occurrence with cardiovascular diseases.

Key challenges include improving target specificity to reduce off-target toxicity, overcoming resistance mechanisms, identifying predictive biomarkers for patient selection, and enhancing drug delivery to solid tumors.^26^ The expanding application beyond oncology, particularly in neurological, cardiovascular, and autoimmune diseases, combined with emerging drug classes targeting specific epigenetic mechanisms, positions epigenetic therapeutics at the forefront of precision medicine approaches for complex diseases.

The clinical landscape of epigenetic therapeutics demonstrates regulatory success, with 13 U.S. FDA-approved drugs targeting key epigenetic regulators (Figure 8A). HDAC inhibitors dominate with 6 approvals and hematological malignancies represent the primary therapeutic application. This concentration reflects the particular sensitivity of blood cancers to epigenetic dysregulation and the accessibility of hematologic targets compared to solid tumors.^497^

**Figure 8:**
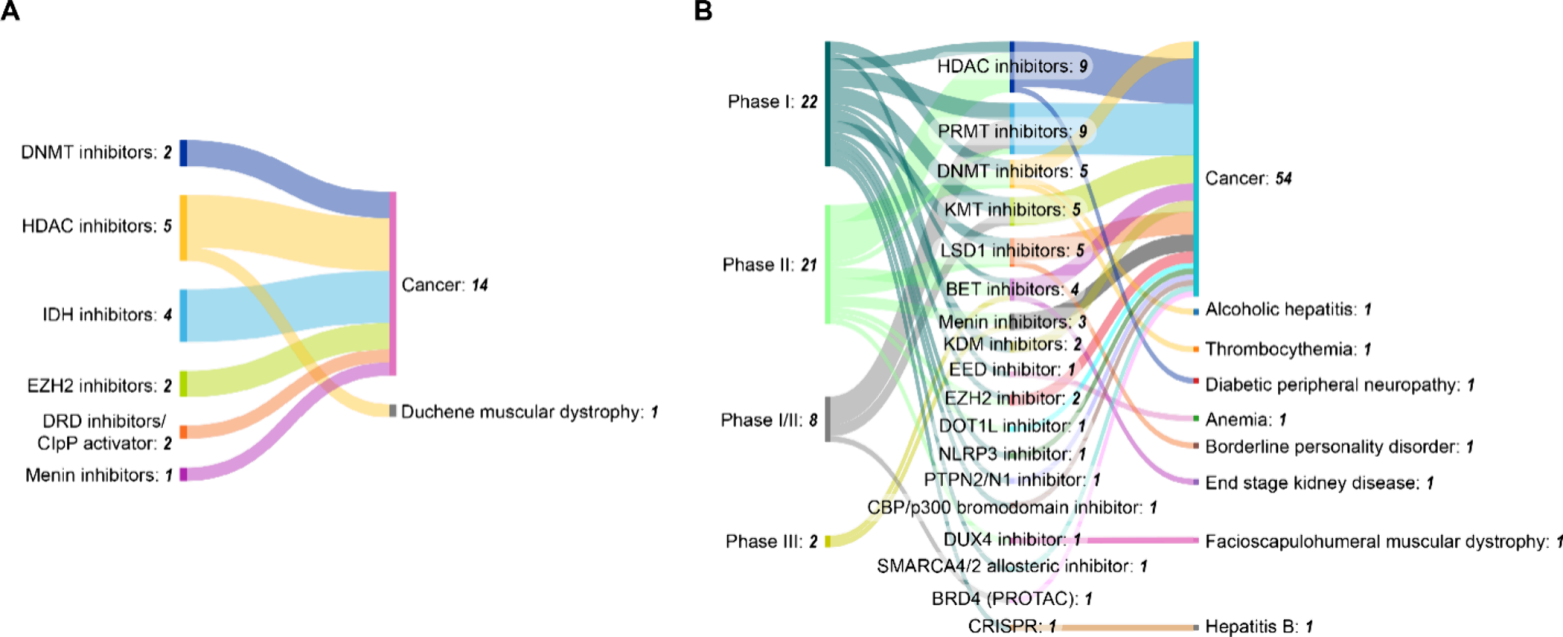
Distribution of (A) FDA approved epigenetic therapeutics and (B) those currently in clinical trials across disease conditions. Does not include combination therapies currently in clinical trials. Each drug/therapy has been counted once in the highest phase trial.

DNMT inhibitors azacitidine and decitabine were among the first approved epigenetic drugs, establishing proof-of-concept for targeting DNA methylation in myelodysplastic syndromes and AML. HDAC inhibitors including vorinostat, romidepsin, and belinostat have shown efficacy across multiple hematologic malignancies, validating histone modification as a therapeutic target.

### Other Therapeutic Modalities and Strategies in Epigenetic Drug Discovery

Discussed briefly are a few therapeutic approaches beyond the existing/well-established epigenetic modulators.^26^

- Dual-action or multiepigenetic modulators and combination strategies

Combine mechanisms targeting multiple epigenetic pathways. These drugs may enhance therapeutic efficacy in complex diseases.^493–495^ This strategy either utilizes multiple targets from same epigenetic mechanisms (e.g., dual inhibitors targeting histone erasers, readers and writers),^498^ multiple targets from two different epigenetic mechanisms (e.g., CM-272),^499,500^ or a combination of targets from epigenetic and nonepigenetic mechanisms^493^ (e.g., CUDC-101).^501^ A recently published article^502^ details the use of computer-aided drug design to identify small molecule inhibitors of multiple methyltransferase-like protein (METTLs, m^6^A methylation writers).

CM-272, a small molecule inhibitor with a quinolone moiety acts as a fairly potent dual reversible inhibitor of DNMTs (DNMT1, DNMT3A) and euchromatic histone-lysine *N*-methyltransferase 2 (EHMT2, also referred to as G9a) with possible use in the treatment of AML, acute lymphoblastic leukemia (ALL), and diffuse large B-cell lymphoma (DLBCL)^499,500^ and eventually expanded beyond to hepatocellular carcinoma (HCC)^503^ and multiple myeloma (MM)^504^ serving as an example of dual-targeting epigenetic modulators utilizing targets from two different epigenetic mechanisms. Other examples include a DNMT/HDAC inhibitor capable of eliciting a viral mimicry response,^505^ defined as “a cellular state in which the reactivation of silenced transposable elements (TEs) leads to the accumulation of immunogenic nucleic acids, triggering innate immune pathways that resemble responses mounted against viral pathogens”^506^ and therefore likely to be useful in combination with immune checkpoint inhibitors.

Examples of dual epigenetic and nonepigenetic targeting agents include CUDC-101, a small molecule inhibitor of HDAC, EGFR, and HER2,^501^ and HH-2853, a small molecule inhibitor of EZH1/2, BRD4, PARP1, and EHMT2 ^507^ among many others.

Integrating epi-drugs with chemotherapy, immunotherapy, and targeted agents show promise for enhanced efficacy.^496^ Some of the most explored combination strategies involve combining U.S. FDA approved DNMT inhibitors azacitidine or decitabine with U.S. FDA approved HDAC inhibitors such as vorinostat and romidepsin or with other investigational HDAC inhibitors such as pracinostat and mocetinostat.^508^ Other combination strategies include designing hybrid molecules containing pharmacophores for more than one epigenetic target.^509^

- CRISPR-based epigenetic therapies

Various CRISPR-based epigenetic therapeutic approaches are being explored including in the treatment of sickle cell anemia,^510^ cancer,^511^ and hepatitis B.^512^ Among these, Tune-401 has reached clinical trials with Phase I study initiated in 2024 (NCT06671093 ^223^).

- PROTACs and protein degraders

The development of PROTACs as a therapeutic modality appears to be occurring at a brisk and continuous pace with several epigenetic PROTACs being pursued. These include PROTACs targeting HDAC6,^513^ bromodomain-containing protein 9 (BRD9)^514,515^ as well as embryonic ectoderm development (EED), a protein that is part of the polycomb repressive complex 2 (PRC2).^516^ Besides these, other epigenetic PROTACs under development include SMARCA2/4 targeting PROTACs (ABCI1 ^517^) as well those targeting other members of the HDAC family such as HDAC3,^518^ HDAC4,^519^ HDAC6 ^520^ and many others. Most epigenetic PROTACs appear to utilize either cereblon targeting ligands such as thalidomide, pomalidomide and their variations or Von-Hippel-Lindau targeting ligands such as VHL1. For instance, compound 17c, a HDAC6 PROTAC utilizes 6-fluorothalidomide to recruit cereblon while the two BRD9 PROTACs CFT8634 and FHD-609 utilize anilino glutarimide and thalidomide, respectively, to also recruit cereblon. The EED PROTAC, ABI1, on the other hand utilized a VHL targeting ligand, VHL1 (Figure S5).

Out of these PROTACs, both BRD9 targeted ones (CFT8634 and FHD-609) reached clinical trials (NCT05355753,^521^ NCT04965753 ^522^). However, both trials were terminated, the former (CFT8634, NCT05355753) due to lack of BRD9 degradation resulting in insufficient efficacy for treating patients with synovial sarcoma^523^ while the latter (FHD-609, NCT04965753) appears to a sponsor decision due to safety concerns (related to a grade 4 QTc prolongation event).^524^ Efforts have also been made toward designing regulated induced proximity targeting chimeras (RIPTACs), a type of heterobifunctional molecule designed for cancer therapy that leads to formation of a ternary complex achieved by targeting two proteins one of which is exclusively expressed in cancer cells while the other is essential for cell survival.^525,526^ RNK-05047 (Ranok Therapeutics (Hangzhou) Co., Ltd.) is a RIPTAC that has entered Phase I/II trials and is actively recruiting individuals (NCT05487170,^527^ Table S3). While the termination of the two clinical trials is disappointing, the development of PROTACs and other protein degraders is only likely to continue and accelerate.^528^

- Antisense oligonucleotides (ASOs)

Antisense oligonucleotides (ASOs) are short single strand RNA or DNA fragments usually comprising of 15–21 nucleotides and synthesized to bind to mRNA to achieve change in protein expression by inhibition of or increasing translation, by causing RNA knockdown by causing degradation of RNA.^529,530^ While ASOs have been around for a while, it is only in the past 10 or so years that development of ASOs as therapeutics has increased with multiple ASOs entering clinical trials.^529–531^ In the area of epigenetics, ASOs are being pursued to target lncRNAs such as Chaserr potentially useful in treating neurological disorders such as epilepsy^532^ or methylation writers such as EZH2 ^533^ in combination with androgen receptors to effectively inhibit growth of castration-resistant prostate cancer cells.^533^

- miRNA mimics

miRNA mimicry involves the use of synthetic double-stranded microRNAs that imitate endogenous miRNA^534^ allowing regulation of gene transcription.^535^ The first miRNA mimic entered clinical trials in the early 2010s^536^ and while there has been continued interest in the development of miRNA mimics, persistent challenges remain.^537^ In the epigenetic landscape, the use of miRNA mimics has been mostly been explored in the treatment of cancer (MRX34, miR-34 mimic, NCT01829971,^538^ NCT02862145;^539^ MesomiR-1, miR-16 mimic, NCT02369198;^540^ INT-1B3, miR-193a mimic, NCT04675996 ^541^) and keloid (MRG-201, miR-29 mimic, NCT03601052 ^542^). The miR-34 clinical trials had to be terminated or withdrawn due to severe immune related adverse events, while the miR-193a trial was terminated due to insufficient funding. MesomiR-1, a type of TargomiR described as nonliving bacterial minicells packaged with miRNA mimic, showed promising results with reasonable safety profile.^543,544^ In 2022, the development of MRG-229, another miR-29 mimic based on MRG-201, was reported with potential use in treating idiopathic pulmonary fibrosis.^545^ While clinical development of miRNA mimics has a long way to go, they nonetheless remain an important avenue for continued exploration.

## Epigenetic Drugs in Clinical Trials

The epigenetic drug clinical trial landscape has expanded dramatically over 25 years, with nearly 2,200 trials registered on clinicaltrials.gov.^546^ Figure 9 shows sustained increase from a single trial in 2000 to just under 200 clinical trials a year in 2024, with notable oscillations reflecting regulatory milestones and market dynamics. Following azacitidine’s U.S. FDA approval in 2004, trial activity demonstrated waxing and waning patterns with overall upward trajectory. This growth pattern indicates sustained pharmaceutical investment despite periodic consolidation phases.

**Figure 9:**
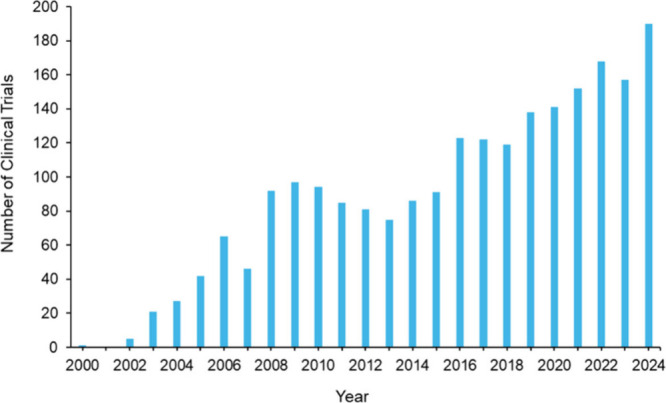
Number of therapeutic epigenetic drug clinical trials by year. Source: CAS, publicly available information from clinicaltrials.gov.^546^ Clinical trials are characterized by first posted date.

Analysis of therapeutic epigenetic clinical trials with respect to phase distribution reveals that Phase II trials dominate at 57%, followed by Phase I (32%) and Phase III (9%) (Figure 10A). This distribution is typical of drug development and reflects the high attrition rate in epigenetic drug development and the exploratory nature of many combination strategies. Similar analysis in terms of trial status (Figure 10B) shows that 36% of all clinical trials over the past 25 years have been completed, while 29% of trials remain in early development (not yet recruiting, recruiting, and active status). The phase-specific status breakdown (Figure 10C) shows that Phase I trials have the highest percentage of completed trials (53%) while Phase III trials demonstrate highest active recruitment (29%). For active trials we see the highest percentages for Phase IV and Phase III trials at 30% and 15%, respectively.

Notably, 62% of trials involve U.S. FDA-approved drugs (Supplementary Figure S6), suggesting extensive label expansion efforts and combination therapy exploration beyond initial indications. The remaining 38% of clinical trials are for newer/novel unapproved drugs. Exemplary nonregulatory approved epigenetic drugs active in the clinical trial pipeline are summarized in Supplementary Table S3.

The clinical development pipeline reveals robust activity with more than 50 ongoing trials across three phases. Phase I and II trials dominate (22 and 21 trials) with DNMT and HDAC inhibitors maintaining a strong clinical presence with 5 and 9 candidates in trials, respectively. However, the clinical landscape shows diversification beyond DNMT and HDAC inhibitors, with LSD1, BET and menin inhibitors being increasingly explored. Additionally, a BRD4 PROTAC and CRISPR based epigenetic therapy are also currently in Phase I (Figure 8B and Table S3). This distribution demonstrates both continued optimization of established targets and expansion into novel epigenetic regulators.

Cancer applications continue to predominate with more than 50 trials, though data suggests encouraging diversification into nononcologic conditions including thrombocythemia, alcoholic hepatitis, anemia, and diabetic peripheral neuropathy. This expansion reflects growing recognition of epigenetic contributions to diverse pathologies beyond cancer.

### Promising Epigenetic Agents in Clinical Development

#### Oncology Agents

**Pelabresib** (**CPI-0610**), an advanced BET inhibitor, reached Phase III trials for myelofibrosis in the MANIFEST-2 study (NCT04603495 ^547^). The U.S. FDA gave Fast Track Designation to pelabresib for myelofibrosis in December 2019.^548^ The progression to Phase III indicates strong Phase II efficacy data with a 35% spleen volume reduction rate when combined with ruxolitinib.^549^ Its mechanism of action involves disrupting the interaction between BET proteins and acetylated chromatin, leading to downregulation of oncogenic transcription.^550^

**Ziftomenib** (**KO-539**), a selective small-molecule inhibitor of the menin-KMT2A protein–protein interaction,^551^ received U.S. FDA Breakthrough Therapy designation in March 2024 for relapsed or refractory NPM1 mutant AML.^552^ In the ongoing KOMET-001 Phase I/II trial (NCT04067336 ^553^), ziftomenib has demonstrated meaningful clinical activity in both NPM1 mutant AML (30% of cases) and KMT2A-rearranged AML (10% of adult cases).^554^ This targeted therapy works by disrupting menin-KMT2A interactions to downregulate specific oncogenes and induce myeloid differentiation.^555^

#### Nononcology Agents

##### Cardiovascular and Metabolic Indications

**Apabetalone** (**RVX-208**), a selective BET inhibitor targeting BD2 domains, is currently in Phase I/II for end-stage kidney disease (NCT03160430 ^556^). The U.S. FDA previously granted Breakthrough Therapy Designation in 2020 for major adverse cardiovascular events (MACE) reduction in high-risk type 2 diabetes patients with coronary artery disease.^557^ Unlike oncology-focused BET inhibitors, apabetalone modulates cardiovascular and renal disease pathways through selective inhibition of BRD4.^558^ This represents a new approach to treating both kidney and cardiovascular diseases through epigenetic modulation, potentially establishing BET inhibition as a new therapeutic approach for chronic metabolic and inflammatory conditions.

**Larsucosterol** (**DUR-928**), an endogenous sulfated oxysterol and first-in-class epigenetic regulator, is in Phase II trials for alcoholic hepatitis (NCT04563026 ^559^). The U.S. FDA granted Breakthrough Therapy Designation for alcoholic hepatitis in 2024.^560^ Larsucosterol functions via DNMT inhibition and modulating lipid metabolism, inflammatory responses, and cell survival pathways.^561^ Phase II results showed a 74% survival rate at day 28 compared to 53% for standard of care in severe alcoholic hepatitis patients.^562^ Larsucosterol was also explored in a Phase 1b clinical study in nonalcoholic steatohepatitis (NASH) patients where it showed a good safety profile and improvement in insulin resistance, liver stiffness, liver enzyme, and biomarkers for liver health.^561^

#### Neuropsychiatric Applications

**Vafidemstat (ORY-2001)** is a selective LSD1 inhibitor that is currently being evaluated in Phase II clinical trials for borderline personality disorder (NCT04932291 ^563^). This represents a novel therapeutic approach to treating psychiatric conditions through epigenetic modulation, specifically targeting lysine-specific demethylase1 (LSD1) to potentially address the underlying neurobiological mechanisms of personality disorders.^564^ The development of vafidemstat for borderline personality disorder highlights the expanding application of epigenetic therapies beyond oncology into complex psychiatric and neurological conditions.

#### Recently Approved Agents: Validating Novel Mechanisms

##### First-in-Class Approvals

**Modeyso** (**Dordaviprone**) received U.S. FDA accelerated approval in August 2025 for H3K27 M mutant diffuse midline gliomas in patients over the age of one.^565^ This first in class drug represents a novel epigenetic approach through dopamine receptor antagonism, leading to integrated stress response activation and epigenetic modulation.^566^ It addresses a critical unmet need in neuro-oncology with a 5-year survival rate of 1%.^567^ Previous/Completed clinical trials have shown that Modeyso had successful tumor uptake with therapeutic intratumoral concentrations.^568^ The ongoing Phase III ACTION study (NCT05580562 ^569^) continues efficacy and safety evaluation.

**Revuforj** (**Revumenib**) gained U.S. FDA approval in November 2024 for relapsed/refractory KMT2A-rearranged AML in adults and pediatric patients.^570^ The AUGMENT-101 trial (NCT04065399 ^571^) demonstrated a 63% overall response rate in KMT2Ar acute leukemia,^572^ representing first U.S. FDA-approved menin inhibitor for cancer treatment.^573^ Ongoing trials for AML (NCT06652438 ^574^) and colorectal carcinoma (NCT05731947 ^575^) explore broader applicability of menin inhibition.

#### Future Perspectives

The clinical pipeline demonstrates maturation of epigenetic therapeutics beyond first-generation DNMT and HDAC inhibitors toward precision-targeted agents. The expansion into nononcology indications such as cardiovascular, metabolic, and neuropsychiatric diseases, validates epigenetic modulation as a broadly applicable therapeutic strategy. With multiple Phase III programs and recent approvals validating novel mechanisms, the field approaches an inflection point where epigenetic drugs may transform treatment paradigms across diverse pathologies.

**Figure 10:**
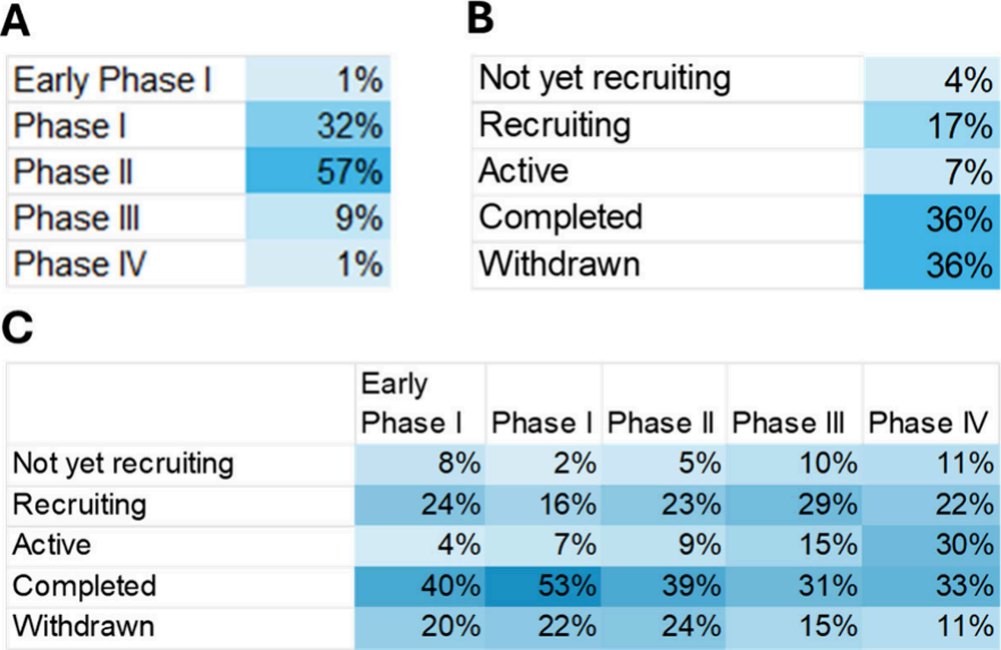
Distribution of epigenetic drug clinical trials in terms of (A) overall clinical trial phase development, (B) overall clinical trial statuses, (C) clinical trial phases compared against statuses. Phase I/II and Phase II/III studies are classified under Phase II and Phase III studies, respectively. Source: CAS, publicly available information from clinicaltrials.gov.^546^

## Epigenetic Biomarkers: Clinical Applications and Future Perspectives

Epigenetic biomarkers are molecular signatures derived from epigenetic modifications that reflect changes in gene expression patterns without altering the DNA sequence. These biomarkers are increasingly recognized for their potential in disease diagnosis, prognosis, and therapeutic decision-making. Their reversible nature and responsiveness to environmental factors make them particularly valuable for understanding complex diseases such as cancer,^576^ neurological disorders,^577^ and cardiovascular diseases.^398^ Temporal analysis of publication indicate a sharp increase starting around 2008–2009 and marked by a few periods of plateauing (2015–2017 and 2023–2024) (Figure 11A). Among the four main epigenetic mechanisms, DNA methylation and ncRNA appear to be the most well-studied consistently, while histone modifications and chromatin remodeling remain comparatively underexplored in this context. (Figure 11A, inset). The sankey diagram (Figure 11B) illustrates the distribution of epigenetic biomarker research across disease categories and mechanism types. Cancer appears to co-occur most extensively with publications related to epigenetic biomarkers by a long margin, reflecting the well-established role of epigenetic dysregulation in oncogenesis and the clinical utility of methylation markers such as MGMT^578^ and VIM^579^ for diagnosis and prognosis. The substantial representation of aging research aligns with recent advances in epigenetic clock development and their applications in biological age assessment.

**Figure 11:**
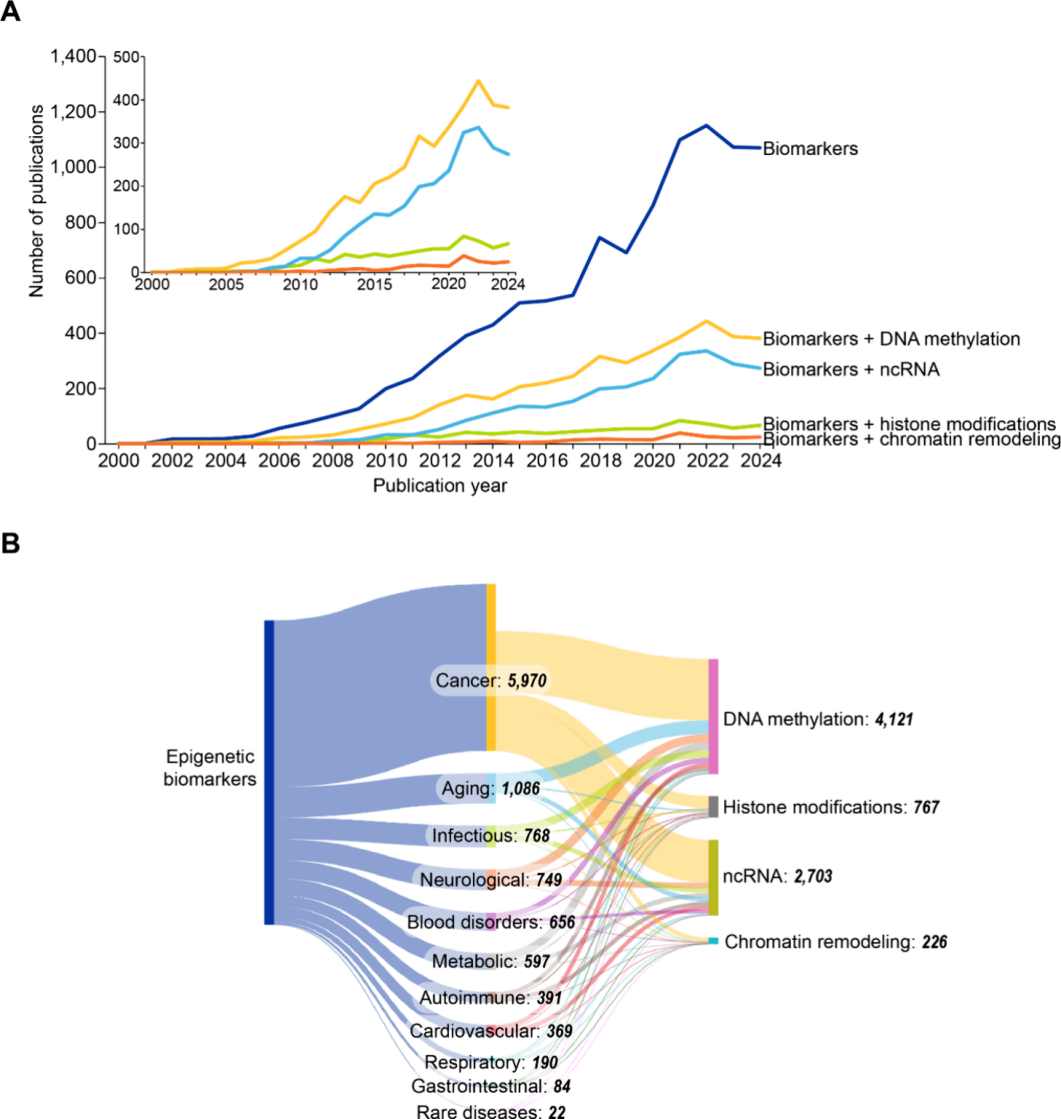
(A) Publication trends for publications related to biomarkers and specific epigenetic mechanisms and (B) their co-occurrences with specific disease types and epigenetic mechanism. Data includes journal articles and patents extracted from the CAS Content Collection for the period 2000–2024.

Epigenetic biomarkers offer distinct advantages:

- Dynamic responsiveness to environmental factors enables real-time disease monitoring;
- Tissue specificity provides organ-specific disease insights;
- Early detection capability precedes clinical manifestation allowing early intervention;
- Noninvasive sampling through blood, urine, and saliva facilitates routine monitoring and improving patient compliance.

Epigenetic biomarkers are broadly categorized based on the type of epigenetic modification they represent.

### DNA Methylation Biomarkers

Aberrant DNA methylation patterns serve as disease hallmarks, with hypermethylation of tumor suppressors (e.g., BRCA1,^580^ p16 ^313^) being a common biomarker of various cancers, while altered BDNF methylation has been linked to depression and schizophrenia.^581^

**SEPT9 methylation** enables noninvasive colorectal cancer screening through blood-based detection (Epi proColon^582^), offering an alternative to colonoscopy. **MGMT methylation** predicts Temozolomide response in glioblastoma, with hypermethylation being associated with better outcomes.^583^ **BRCA1 methylation** serves as diagnostic and prognostic marker associated with increased risk of breast^584^ and ovarian cancer.^585^ Notably, DNA methylation co-occurs most predominantly with epigenetic biomarker research, likely due to its chemical stability, established detection methodologies, and direct clinical translation through U.S. FDA-approved tests.

### Histone Modification Biomarkers

Histone modifications influence chromatin structure and gene expression and specific histone marks are associated with disease states. The relatively modest co-occurrence of histone modifications (Figure 11B) suggests untapped potential, particularly given their dynamic nature and responsiveness to therapeutic interventions. However, challenges remain such as the highly labile nature of histone modifications and their susceptibility to enzymatic degradation causing rapid signal loss within minutes of sample collection. Current protocols require specialized preservation buffers containing protease, deacetylase, and demethylase inhibitors, complicating routine clinical implementation.

**Loss of histone H4 acetylation** predicts poor prognosis in certain cancers,^586^ whereas **increased acetylation at lysine 12 (H4K12)** is a novel biomarker for Alzheimer’s.^587^ Beyond acylation, other post-translational modifications on histones offers additional biomarker potential. H3 lysine 27 trimethylation (**H3K27me3**) reflects PRC2 dysregulation and correlates with poor prognosis in prostate^588^ and bladder cancers.^589^ **Histone H3/H4 citrullination** is linked to the production of anticitrullinated protein antibodies (ACPAs), a hallmark of rheumatoid arthritis enabling rheumatoid arthritis diagnosis and monitoring.^590,591^ **Crotonylation of** H2BK12 **(H2BK12cr)** in peripheral blood mononuclear cells (PBMCs) is significantly elevated in colorectal cancer patients,^592^ while serum **histone succinylation** shows strong correlations with tumor type, stage, and prognosis, enabling rapid pan-cancer screening through a single-tube blood test.^593^ Additionally, increased histone **lactylation at H4K5 (H4K5lac)** in PBMCs and breast cancer tissues correlates with serum lactate and CEA levels, suggesting its potential role in diagnosis and therapeutic strategies.^594^

### ncRNA Biomarkers

ncRNAs, including miRNAs like miR-21 ^595^ and miR-155 ^596^ are overexpressed in various cancers and serve as diagnostic and prognostic biomarkers, often provide accessible disease markers through bodily fluids.^597^ The substantial ncRNA co-occurrence with epigenetic biomarker research reflects growing interest in circulating miRNAs and lncRNAs as minimally invasive biomarkers (Figure 11B).

**miR-21 overexpression** is associated with tumor growth, invasion, metastasis, and chemotherapy resistance across multiple cancers including breast, lung, and colorectal cancer. **Elevated miR-208a levels** serves as a biomarker for acute myocardial infarction (heart attack).^598^ **HOTAIR lncRNA overexpression** is associated with metastasis and poor prognosis in breast,^599^ colorectal,^600^ and pancreatic cancers.^601^

### Chromatin Remodeling Biomarkers

Chromatin remodeling patterns predict therapeutic resistance, with open chromatin regions in drug-resistant cancer cells being used to predict treatment failure.^602^ Similar to histone modifications, the relatively low co-occurrence of chromatin remodeling suggests untapped potential.

The future of epigenetic biomarkers lies in the development of robust, high-throughput technologies and integrative approaches. Multiomic integration combining epigenetic, genomic, transcriptomic, and proteomic data will enhance biomarker discovery and validation. Noninvasive detection of epigenetic biomarkers in bodily fluids will revolutionize early diagnosis and monitoring. Advanced computational tools are expected to aid in identifying complex epigenetic signatures for patient stratification. CRISPR-based epigenome editing may enable manipulation of disease-associated epigenetic marks for therapeutic purposes, potentially transitioning biomarkers from diagnostic to therapeutic tools.

While challenges remain including standardization and validation, technological advances and clinical implementation strategies are establishing epigenetic biomarkers as cornerstone components of precision medicine, offering unprecedented opportunities for early detection, prognostic assessment, and therapeutic guidance across diverse pathologies.

### Emerging Technologies in Epigenetic Research

Technological advances are revolutionizing epigenetic research, enabling precise, scalable investigations into epigenetic modification mechanisms and therapeutic applications. These innovations drive discoveries in gene regulation, disease pathogenesis, and drug development across multiple research domains. In Table S4 we have summarized some of the most exciting and transformative technologies in epigenetic research, highlighting single-cell resolution methods, multiomics integration, and precision editing tools that complement established techniques shown in Figure 12.

**Figure 12:**
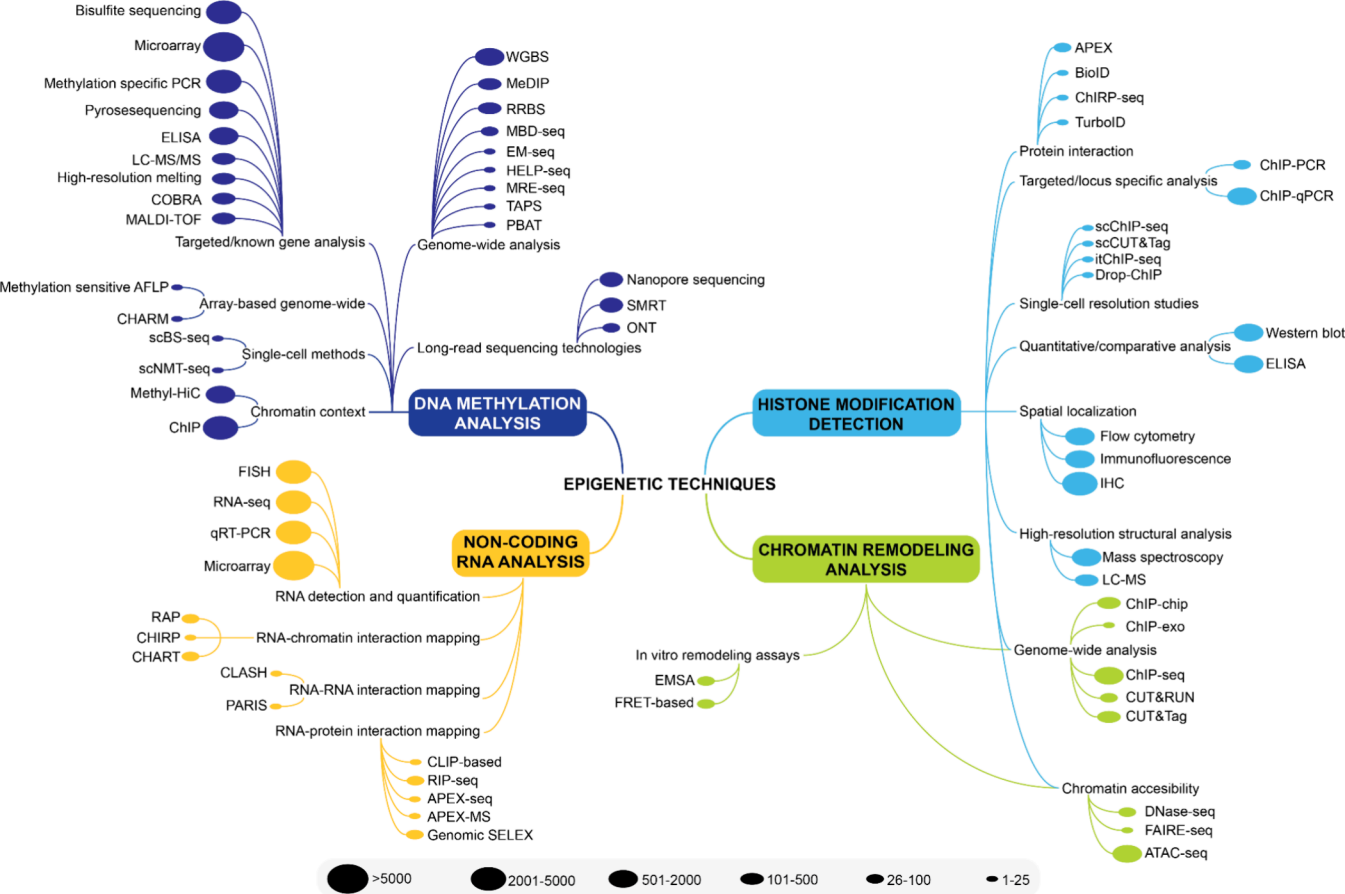
Comprehensive overview of epigenetic publications organized by key epigenetic analytical techniques. The size of the ellipse corresponds to number of publications (journal and patents) extracted from the CAS Content Collection for the period 2000–2024.

The current epigenetic research landscape appears to be focused on four major analytical domains (Figure 12): DNA methylation analysis, histone modification detection, ncRNA analysis, and chromatin remodeling analysis. Our analysis indicates that DNA methylation analysis methods are led by whole-genome bisulfite sequencing (WGBS) and targeted approaches like methylated immunoprecipitation (MeDIP)^603^ and reduced representation bisulfite sequencing (RRBS),^604^ reflecting the maturity of methylation research. Histone modification detection shows strong representation of ChIP-seq variants, with emerging single-cell methods (scChIP-seq, scCUT&Tag) indicating technological evolution toward higher resolution analysis, revealing cell-to-cell variability in cancer, immune responses, and development.^147,166,168,225,605–607^ Single-cell epigenomics addresses limitations of bulk methods that average signals across heterogeneous cell populations.

Multiomics integration combines epigenomic data with transcriptomics, proteomics, and metabolomics using technologies like CUT&RUN^608^ and Hi-C integration,^609^ providing holistic cellular function views.^610,611^ These approaches enable comprehensive pathway analysis and biomarker discovery in complex diseases.

Epigenome editing tools, particularly CRISPR/dCas9-based systems fused with epigenetic effectors (DNMTs, TETs, HDACs), enable targeted modification investigation without DNA sequence alteration.^612,613^ Alternative platforms include TALE^614^ and zinc-finger proteins for locus-specific targeting.^615,616^

Advanced imaging techniques including super-resolution microscopy (STORM,^617^ PALM^618^) and live-cell fluorescent probes enable real-time chromatin dynamics observation,^619,620^ complementing sequencing-based approaches^159^ shown in Figure 11. Long-read sequencing technologies (PacBio, Oxford Nanopore) provide direct modification detection without chemical conversion, addressing limitations in repetitive genomic regions.^621,622^

The technological evolution from traditional methods to emerging approaches demonstrates the field’s progression toward higher resolution, greater precision, and enhanced clinical relevance, positioning epigenetic research for significant therapeutic breakthroughs.

## Ethical, Legal, and Social Implications

Epigenetics has the potential to transform our understanding of health and disease, but it also raises significant ethical, legal, and social challenges.^623–628^ Addressing these challenges requires a proactive and collaborative approach that balances the benefits of epigenetic research with the need to protect individual rights and promote social justice. By developing robust ethical guidelines, legal protections, and public engagement strategies, we can harness the power of epigenetics to improve health and well-being while minimizing potential harms.^623–628^

### Ethical Considerations

#### Privacy and Discrimination

Epigenetic information, like genetic data, is highly personal and can reveal sensitive information about an individual’s health, lifestyle choices, and environmental exposures requiring robust privacy protections. For instance, epigenetic markers predicting disease risk could enable discrimination by health insurers through premium increases or coverage denial. Similarly, the ability to detect epigenetic modifications from past behaviors raises concerns about retroactive health assessments and employment discrimination.

#### Individual Responsibility and Transgenerational Impact

Epigenetic research highlights how lifestyle choices (e.g., smoking, diet) can influence gene expression. This could lead to blaming individuals for their health conditions, ignoring broader social and environmental determinants of health. Epigenetic changes induced by environmental factors can sometimes be passed to future generations. This raises ethical concerns about the long-term consequences of interventions that alter epigenetic markers.

### Legal Framework Requirements

#### Regulatory Oversight

The proliferation of direct-to-consumer epigenetic testing kits raises concerns about accuracy, interpretation, and regulation. Epigenetic biomarkers are increasingly used in diagnostics and personalized medicine. Legal frameworks must ensure that these applications are evidence-based and ethically sound while preventing premature commercialization of unproven technologies.

#### Liability and Intellectual Property

Epigenetic modifications linked to environmental exposure raise complex liability questions regarding responsibility, for instance should toxin-induced harm be the responsibility of employers, manufacturers, or governments? Potential legal accountability for epigenetic harm to fetuses through parental or third-party negligence or environmental exposures would require careful consideration. The commercialization of epigenetic research, such as biomarkers or therapies, raises questions about patenting and ownership, with balancing innovation with public access to epigenetic technologies being a key legal challenge.

### Social Implications

#### Public Understanding and Health Equity

Limited public understanding of epigenetics enables misconceptions and exploitation through unproven therapies including overhyped claims marketed as ″epigenetic diets″. Comprehensive education and robust scientific communication are essential to address this issue and to improve informed decision-making.

Epigenetic research linking environmental exposures to disease may inadvertently contribute to stigmatization of certain communities or populations, particularly those exposed to high levels of pollution or toxins. Ensuring equitable access to epigenetic therapies and preventing exacerbation of health disparities requires deliberate policy interventions addressing socioeconomic barriers.

#### Implementation Strategies

Effective governance requires clear ethical guidelines for epigenetic data collection, use, and sharing; updated legal frameworks addressing unique challenges of epigenetic information; public engagement fostering trust and informed consent; and international cooperation ensuring consistent standards across jurisdictions. By developing robust protections while promoting responsible innovation, society can harness epigenetics’ transformative potential while safeguarding individual rights and promoting health equity.^623–628^

## Future Directions and Challenges

Epigenetics has transformed our understanding of gene-environment interactions and their roles in disease pathogenesis, including cancer, neurodegenerative diseases, and metabolic disorders and has opened new avenues for research and therapy including exploring their potential in personalized medicine. However, the field remains in relative infancy with fundamental questions unanswered. Future progress depends on technological innovation, clinical translation, and addressing complex ethical and methodological challenges.

### Technological Advances Driving Discovery

- Single-cell technologies are revolutionizing the study of epigenetic heterogeneity within tissues and cell populations allowing researchers to map epigenetic landscapes at unprecedented resolution, revealing cell-specific regulatory mechanisms. Spatial epigenomics further preserves tissue architecture, providing context-dependent regulatory insights essential for understanding development and disease progression.
- Advances in next-generation sequencing (NGS) and third-generation sequencing (e.g., nanopore sequencing) are enabling genome-wide profiling of DNA methylation, histone modifications, and chromatin accessibility. These technologies will facilitate the identification of novel epigenetic biomarkers and therapeutic targets.
- CRISPR-Cas9 and related technologies are being adapted for precise editing of epigenetic marks, such as DNA methylation and histone modifications. These tools hold promise for functional studies and therapeutic applications, such as reactivating silenced tumor suppressor genes and modulation of disease-associated regulatory elements.

### Clinical Translation and Precision Medicine

- Epigenetic modifications, such as DNA methylation patterns and miRNA profiles, are being developed as biomarkers for early disease detection, prognosis, and treatment response. For example, epigenetic biomarkers are being explored for cancer screening and monitoring.
- Beyond first-generation epigenetic drugs such as DNMT inhibitors and HDAC inhibitors, already in use for certain cancers, next-generation therapies are likely to include targeted epigenome editing, miRNA-based treatments, ASOs, PROTACs and other protein degraders, and combination strategies.
- Epigenetic profiling can help tailor treatments to individual patients by leveraging their unique epigenetic signatures allowing optimization of treatment selection and dosing and improving efficacy while minimizing adverse effects.

### Mechanistic Understanding and Functional Validation

- Despite remarkable progress in cataloging epigenetic modifications across diverse biological contexts, significant gaps remain in our mechanistic understanding of how these modifications are established, maintained, and erased. Future research must prioritize the functional characterization of these complexes and their context-dependent roles in gene regulation.

### Epitranscriptomics and ncRNA Regulation

- Recent studies have demonstrated that lncRNAs are extensively modified by m^6^A, with these modifications influencing their stability, localization, and protein interactions and potentially play a role in diseases such as cancer.^629–631^ The role of RNA modifications in miRNA biogenesis and function has revealed additional layers of regulatory complexity^632^ with modifications in other ncRNAs suggesting that epitranscriptomic regulation extends across the entire spectrum of regulatory RNAs.
- The identification of disease-associated changes in RNA modification patterns may provide new diagnostic biomarkers and therapeutic targets and understanding the crosstalk between DNA modification and RNA modifications will be essential for developing comprehensive models of gene regulation.

Targeting the epitranscriptomic machinery using small molecule inhibitors^633^ and incorporating epitranscriptomic modifications to enhance stability, specificity, and efficacy of RNA-based therapeutics are viable strategies being pursued.

### Interdisciplinary Integration and Data Management

- Epigenetics intersects with fields such as genetics, bioinformatics, environmental science, and social sciences. Understanding gene-environment interactions and their impact on health and disease requires collaboration across these domains, integrating molecular mechanisms with population-level health outcomes.
- Integrating epigenomic data with transcriptomic, proteomic, and metabolomic data will provide a more comprehensive understanding of gene regulation and cellular function. Machine learning and artificial intelligence (AI) will play a key role in analyzing and interpreting these complex data sets, identifying potential patterns invisible to traditional analyses and predicting therapeutic responses but will likely require careful validation and interpretation.
- The generation of large-scale epigenomic data sets demand robust computational infrastructure and presents challenges in data storage, analysis, interpretation, and sharing. Developing standardized protocols and computational tools including interoperable databases, and cloud-based platforms will be critical for reproducible research and meta-analyses across studies contributing to advancing the field.

### Persistent Challenges

Despite its potential, epigenetics faces several challenges that must be overcome. The dynamic and complex interplay between genetic, epigenetic, and environmental factors complicates the identification of causal mechanisms. Understanding context-specific effects and tissue-specific regulation requiring sophisticated experimental designs and analytical approaches to disentangle remains a major challenge. Current technologies for mapping epigenetic modifications are not yet fully comprehensive or cost-effective, limiting accessibility. Single-cell methods suffer from technical noise and dropout, while spatial techniques sacrifice resolution for coverage. Developing more robust, scalable, and cost-effective methods remains a priority. Variability in experimental protocols, data processing pipelines and analysis methods can lead to inconsistent results. Establishing standardized guidelines and best practices are critical to improve reproducibility and reliability.

## CONCLUSIONS

The landscape of epigenetic research has undergone a remarkable transformation over the past decade, evolving from a specialized area of molecular biology into a mainstream biomedical discipline with profound implications for human health and therapeutic intervention. This remarkable expansion has been driven by a complex interplay and convergence of scientific breakthroughs, technological innovations, regulatory evolution and economic incentives that have transformed our understanding of gene regulation and its implications for human health. The potential pleiotropic effects of epigenetic therapies^634^ with applications across multiple diseases have altered cost-benefit calculations and require novel approaches to value assessment unlike most conventional drugs. Technological innovations have served as critical enablers of epigenetic research growth, with NGS technologies revolutionizing the scale and precision of epigenomic analysis. The development of ChIP-seq and bisulfite sequencing technologies has allowed comprehensive analysis of histone modifications and DNA methylation patterns, respectively. Recent and ongoing cutting-edge innovations such as single-cell epigenomics and spatial epigenomics continue to push the field forward.

The clinical translation of epigenetic research has achieved remarkable success, with 13 U.S. FDA-approved drugs validating epigenetic targets as therapeutically viable. The more than 35 ongoing clinical trials across multiple development phases indicate sustained investment and confidence in epigenetic therapeutics, with encouraging diversification beyond oncology, with trials spanning metabolic, neurological, inflammatory, and rare diseases. The potential for epigenetic age clocks and other aging biomarkers to find applications in clinical practice and consumer health markets has generated additional commercial interest. The surge in interest in CRISPR-based therapies has meant the concomitant development of epigenome editing technologies, including dCas9-based systems and base editors, all of which are poised to open new possibilities for therapeutic intervention and research applications.

The continued emergence of environmental epigenetic research reflects growing recognition that environmental factors, such as diet, stress, toxins, and lifestyle choices can induce heritable epigenetic changes with transgenerational implications.The integration of environmental exposure data with individual epigenetic profiles enables personalized risk assessment and intervention strategies, representing a fundamental shift toward preventive, precision medicine approaches.

The future of epigenetics is bright, with immense potential for advancing our understanding of biology and improving human health. However, realizing this potential will require overcoming significant challenges, including technological limitations, ethical concerns, and the complexity of epigenetic regulation. By fostering interdisciplinary collaboration, investing in innovative technologies, and addressing ethical and social implications, the field of epigenetics can continue to make groundbreaking discoveries and translate them into meaningful clinical and societal benefits.

## Supplementary Material



## Notes

Detailed description of methodology, figures resulting from data analysis of the CAS Content Collection data (Figures S1–S4), structures of epigenetic PROTACs (Figure S5), and clinical trial data (Figure S6), table listing notable patents related to epigenetics (Table S1), table listing approved epigenetic drugs (Table S2), table listing information related to epigenetic drugs currently in clinical trials (Table S3), table summarizing emerging technologies in epigenetics (Table S4) (PDF)

## Abbreviations

ABCA1: ATP binding cassette subfamily A member 1
ACE: angiotensin I converting enzyme
ACPA: anticitrullinated protein antibody
AHRR: aryl hydrocarbon receptor repressor
AI: artificial intelligence
ALKBH5: alkB homologue 5, RNA demethylase
ALL: acute lymphoblastic leukemia
AML: acute myeloid leukemia
APC: APC regulator of Wnt signaling pathway
ARID1A: AT-rich interaction domain 1A
ATAC-seq: assay for transposase-accessible chromatin using sequencing
BDNF: brain derived neurotrophic factor
BET: Bromodomain and extra-terminal domain
BPA: bisphenol A
BRCA1: BRCA1 DNA repair associated
CDKN2A: cyclin dependent kinase inhibitor 2A
CHD: chromodomain helicase DNA
ChIP-seq: chromatin immunoprecipitation sequencing
CLL: chronic lymphocytic leukemia
COMT: catechol-O-methyltransferase
CRISPR: clustered regularly interspaced short palindromic repeats
CUT&RUN: cleavage under targets and release using nuclease
dCas9: direct catalytically inactive Cas9
DLBCL: diffuse large B-cell lymphoma
DNMT: DNA methyltransferases
eNOS: nitric oxide synthase 3
F2RL3: F2R like thrombin or trypsin receptor 3
FDA: Food and Drug Administration
FMR1: fragile X messenger ribonucleoprotein 1
FTO: fat mass- and obesity-associated protein
gRNA: guide RNA
H3K27me3: histone H3 lysine 27 trimethylation
HAT: histone acetyltransferases
HCC: hepatocellular carcinoma
HDAC: histone deacetylases
HDM: histone demethylases
HMT: histone methyltransferases
HOTAIR: HOX transcript antisense intergenic RNA
IBD: inflammatory bowel disease
IDH: isocitrate dehydrogenase
ISWI: imitation switch
KCNQ1: potassium voltage-gated channel subfamily Q member 1
KDM: histone lysine demethylase
KRAS: c-*K*_i_-Ras
lncRNA: long noncoding RNA
m^6^A: N6-methyladenosine
MACE: major adverse cardiovascular event
MECP2: methyl-CpG binding protein 2
MeDIP: methylated immunoprecipitation
MeRIP-seq: m^6^A specific RNA immunoprecipitation sequencing
MGMT: O-6-methylguanine-DNA methyltransferase
miCLI: m^6^A individual-nucleotide resolution cross-linking and immunoprecipitation
miRNA: microRNA
MM: multiple myeloma
MS: multiple sclerosis
NAFLD: nonalcoholic fatty liver disease
NASH: nonalcoholic steatohepatitis
ncRNA: noncoding RNA
NGS: next-generation sequencing
NMPA: National Medical Products Administration
PALM: photoactivated localization microscopy
PBMC: peripheral blood mononuclear cell
PINK1: PTEN-induced kinase 1
piRNA: PIWI-interacting RNA
PKMT: protein lysine methyltransferase
PMDA: Pharmaceuticals and Medical Devices Agency
PPARGC1A: PPARG coactivator 1 alpha
PPARγ: peroxisome proliferator activated receptor γ
PRC2: polycomb repressive complex 2
PRMT: protein arginine methyltransferase
PROTAC: proteolysis targeting chimera
RA: rheumatoid arthritis
RNA: ribonucleic acid
RRBS: reduced representation bisulfite sequencing
SCN5A: sodium voltage-gated channel alpha subunit 5
SEPT9: septin-9
SHANK3: SH3 and multiple ankyrin repeat domains 3
siRNA: small interfering RNA
SLE: systemic lupus erythematosus
snoRNA: small nucleolar RNA
snRNA: small nuclear RNA
SREBF1: sterol regulatory element binding transcription factor 1
STORM: stochastic optical reconstruction microscopy
SWI/SNF: switch/sucrose nonfermentable
T1D: type 1 diabetes
T2D: type 2 diabetes
TALE: transcription activator-like effector
TET: ten-11 translocation
TXNIP: thioredoxin interacting protein
USD: United States Dollar
UTR: untranslated regions
VIM: vimentin
WGBS: whole-genome bisulfite sequencing
XIST: X inactive specific transcript
