# Supplementary material for: The Future of Epigenetics: Emerging Technologies and Clinical Applications
Source: ACS Pharmacol Transl Sci. 2026 Feb 25;9(3):506–44. doi: 10.1021/acsptsci.5c00729 (PMC12993786; doi:10.1021/acsptsci.5c00729)
Supplement: Supplementary file 1 [file pt5c00729_si_001.pdf]

Supporting Information for

The Future of Epigenetics: Emerging Technologies and Clinical Applications

Kavita A Iyer,[1†] Rumiana Koynova-Tenchov,[1†] Janet M Sasso,[1] Trupti Thite,[2] Yi Deng,[1] Qiongqiong Angela Zhou[1]*

[1]CAS, a Division of the American Chemical Society, Columbus, Ohio 43210, United States

[2]ACS International India Pvt. Ltd., Pune 411044, India

[†]Co-first authors

*corresponding author; Email: qzhou@cas.org

- Methods
- Figures S1-S6
- Table S1-S4
- References

Methods

Search query: The search query crafted by CAS subject matter experts, adjusted iteratively to minimize noise while ensuring maximum coverage of the field, consisted of epigenetic? or epigenomic? resulting in ~128K documents over a period of 2000-2025.

Data extraction: Data was extracted from the CAS Content Collection and consisted of document, CAS indexed concept and patent activity data. In terms of document data for journal publications, information included title, abstract, year of publication, number of citations, the first author's affiliation, the country/region of the affiliation. For patent publications, information pertaining to patent offices and patent assignees were also included in addition to other pertinent information. Concept data consisted of CAS indexed terms and their roles associated with publications. Finally, patent activity data consisted of comprehensive information about individual patents in patent families and their filing across more than 90 patent offices worldwide. Data for 2025 is partial and includes data for January-March.

Data analysis:

Leading research organizations were identified on the basis of volume of research output, number of citations, and a combination of both. Leading patent assignees were identified based on volume of patents filed and separated into commercial and non-commercial patent assignees. Leading journal publishers were identified on the combination of volume of research published and the average number of citations per publication. Geographical information (country/region) and organizational affiliation was that of the first author or the first patent assignee for journal and patent publications, respectively. In general, data analysis was performed by searching for keywords in the title, abstract, and CAS indexed terms associated with publications and included multiple synonyms as well as abbreviations.

Data visualization: Figures and images were created using a combination of Tableau, Microsoft Excel, and Adobe illustrator. Additionally, www.BioRender.com was also utilized for creating some of the schematic illustrations and has been specified in Figure captions.

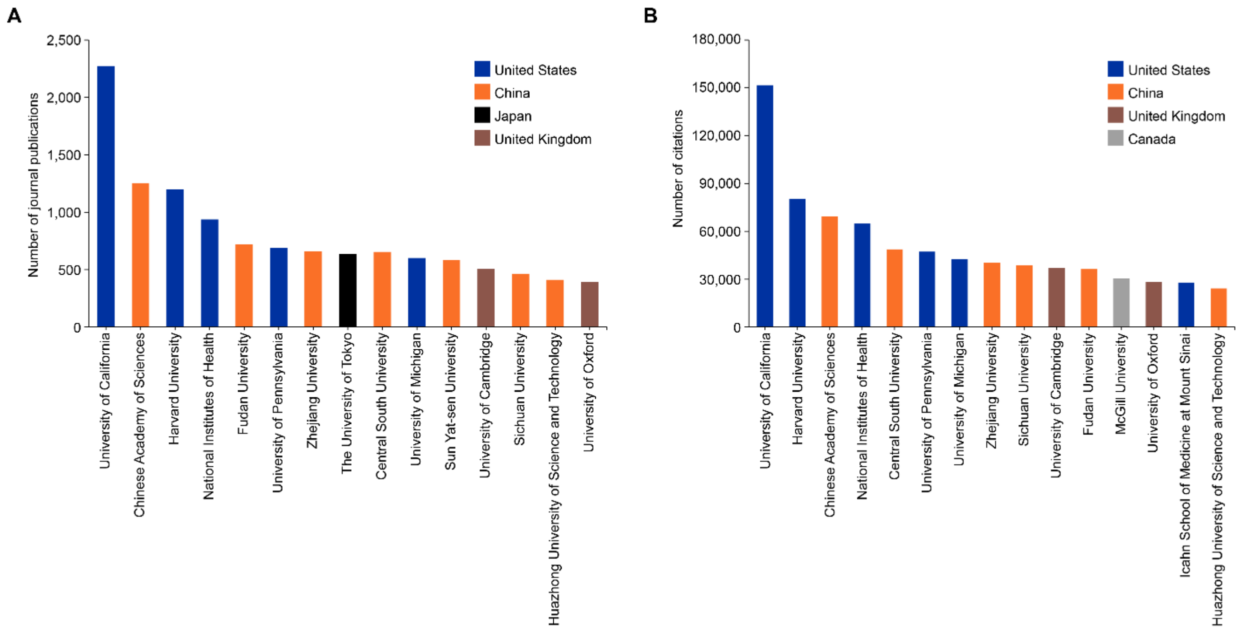

Figure S1. Leading research organizations ranked by (**A**) volume of research output (number of journal publications) and (**B**) impact (number of citations) actively pursuing research opportunities in the area of epigenetics. Data includes journal publications from the CAS Content Collection for the period 2004-2024.

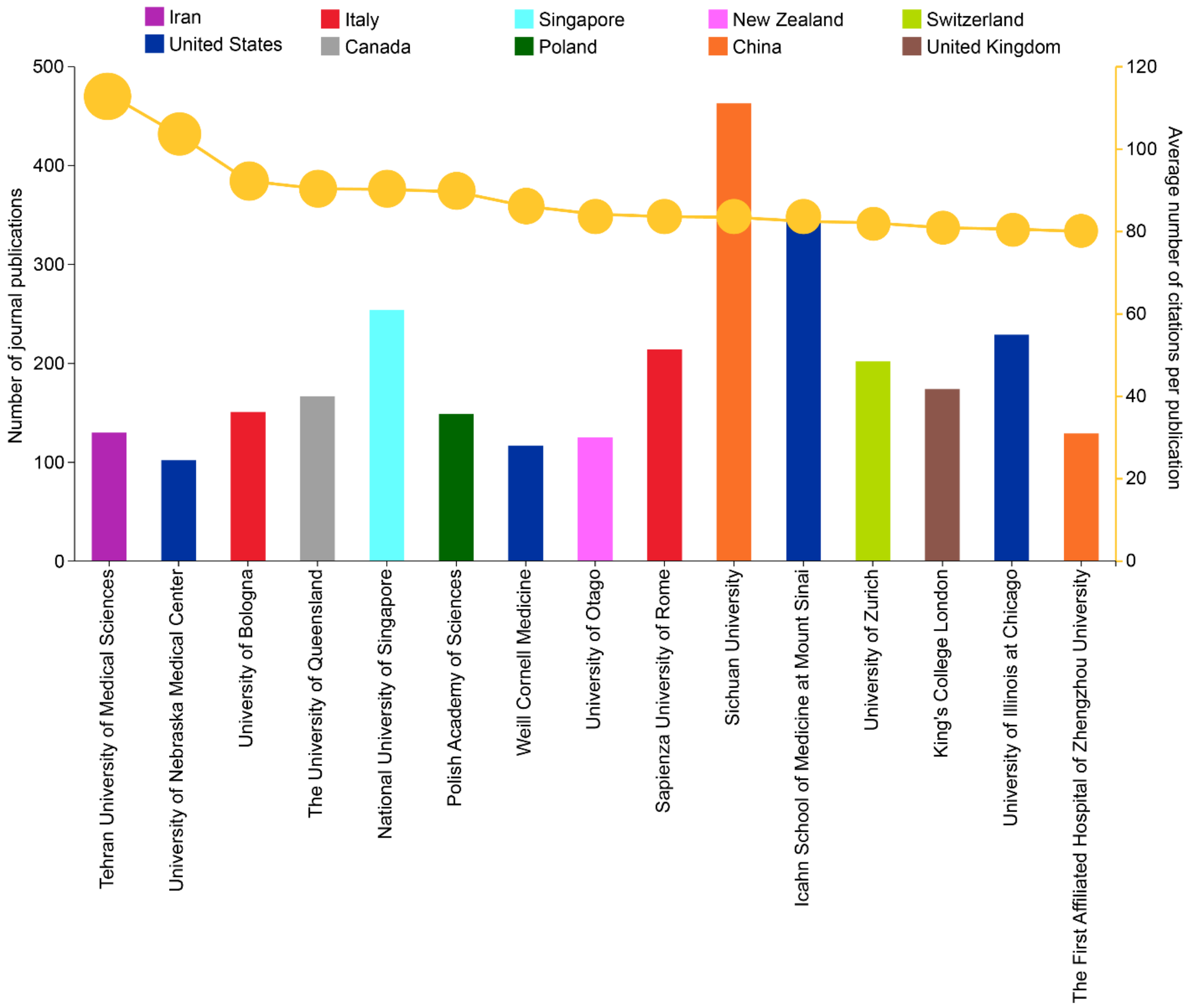

Figure S2. Leading research organizations ranked by a combination of their volume of research output (number of journal publications) and impact (average number of citations per publication) actively pursuing research opportunities in the area of epigenetics. Data includes journal publications from the CAS Content Collection for the period 2004-2024.

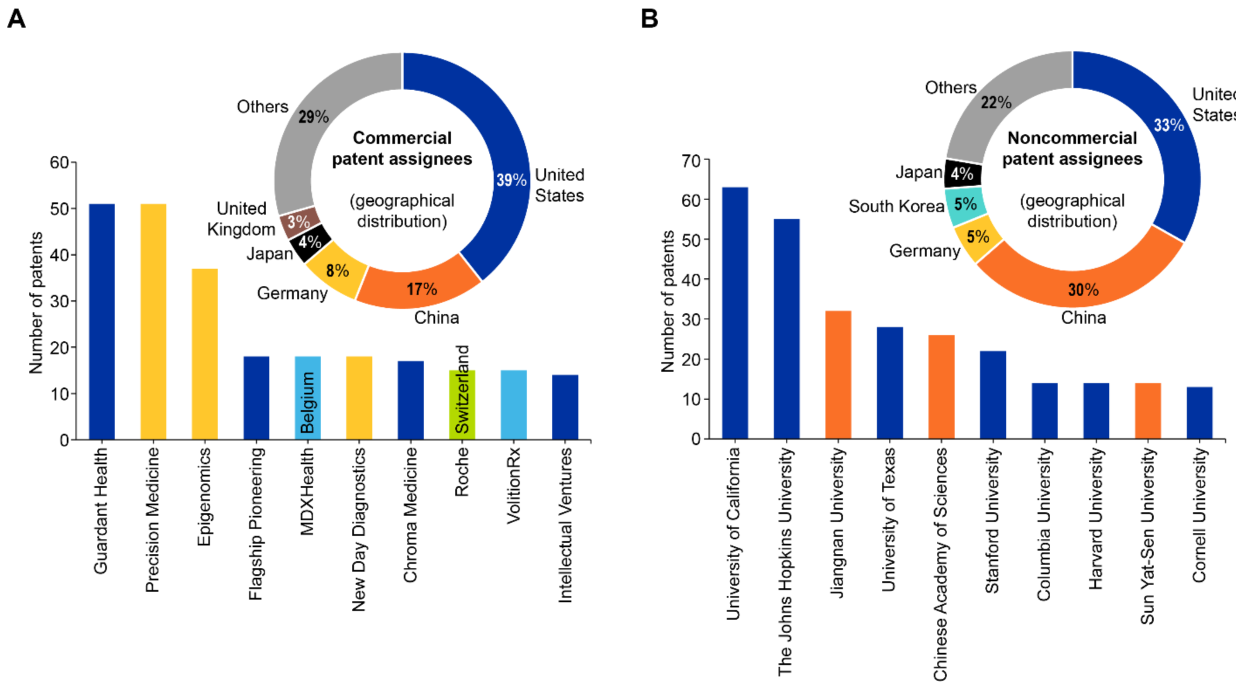

Figure S3. Leading patent assignees divided by (**A**) commercial and (**B**) noncommercial entities actively pursuing research opportunities in the area of epigenetics. Data includes patent publications from the CAS Content Collection for the period 2004-2024.

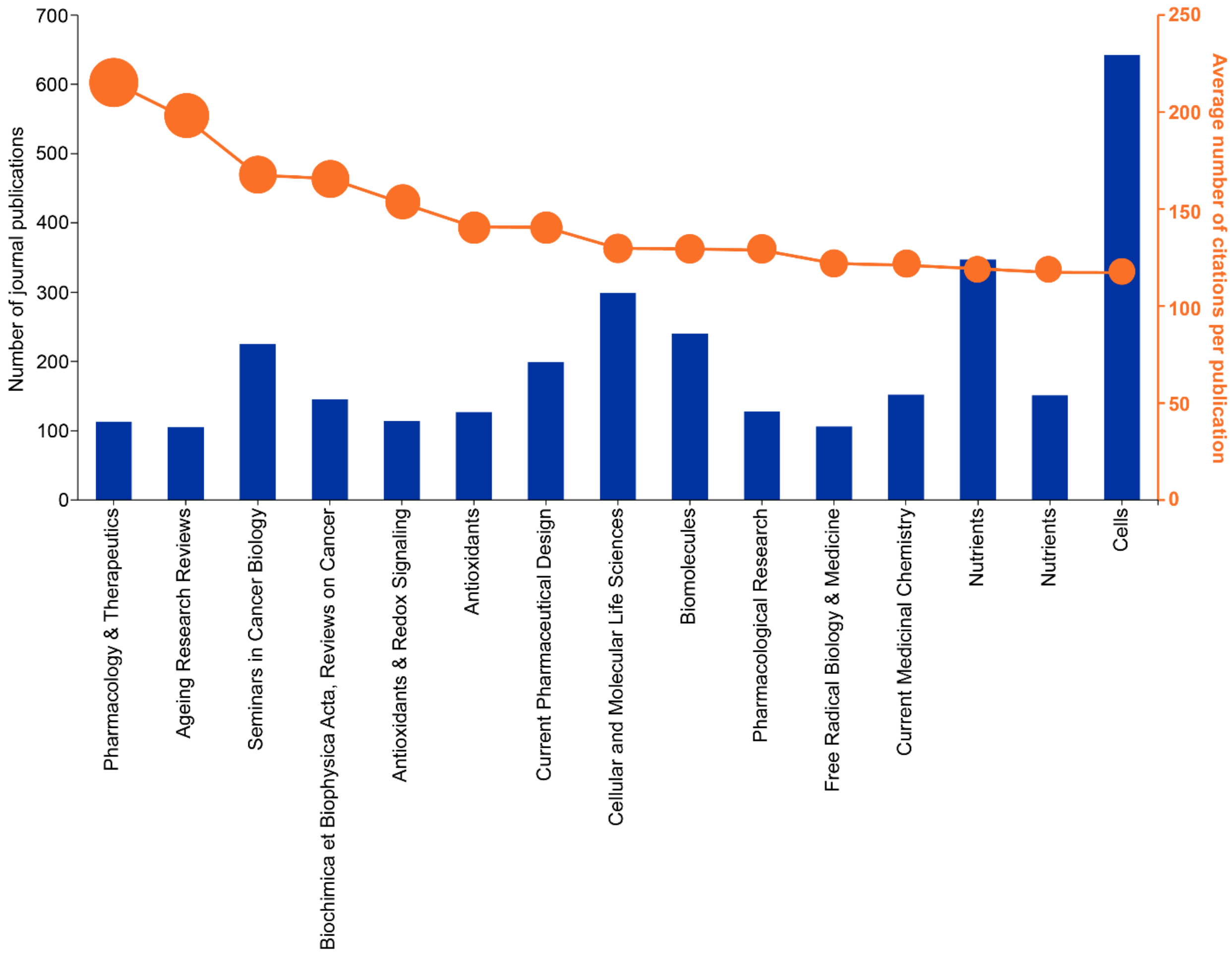

Figure S4. Leading journal publications ranked by a combination of the volume of research published (number of journal publications) and impact (average number of citations per publication) in the area of epigenetics. Data includes journal publications from the CAS Content Collection for the period 2004-2024.

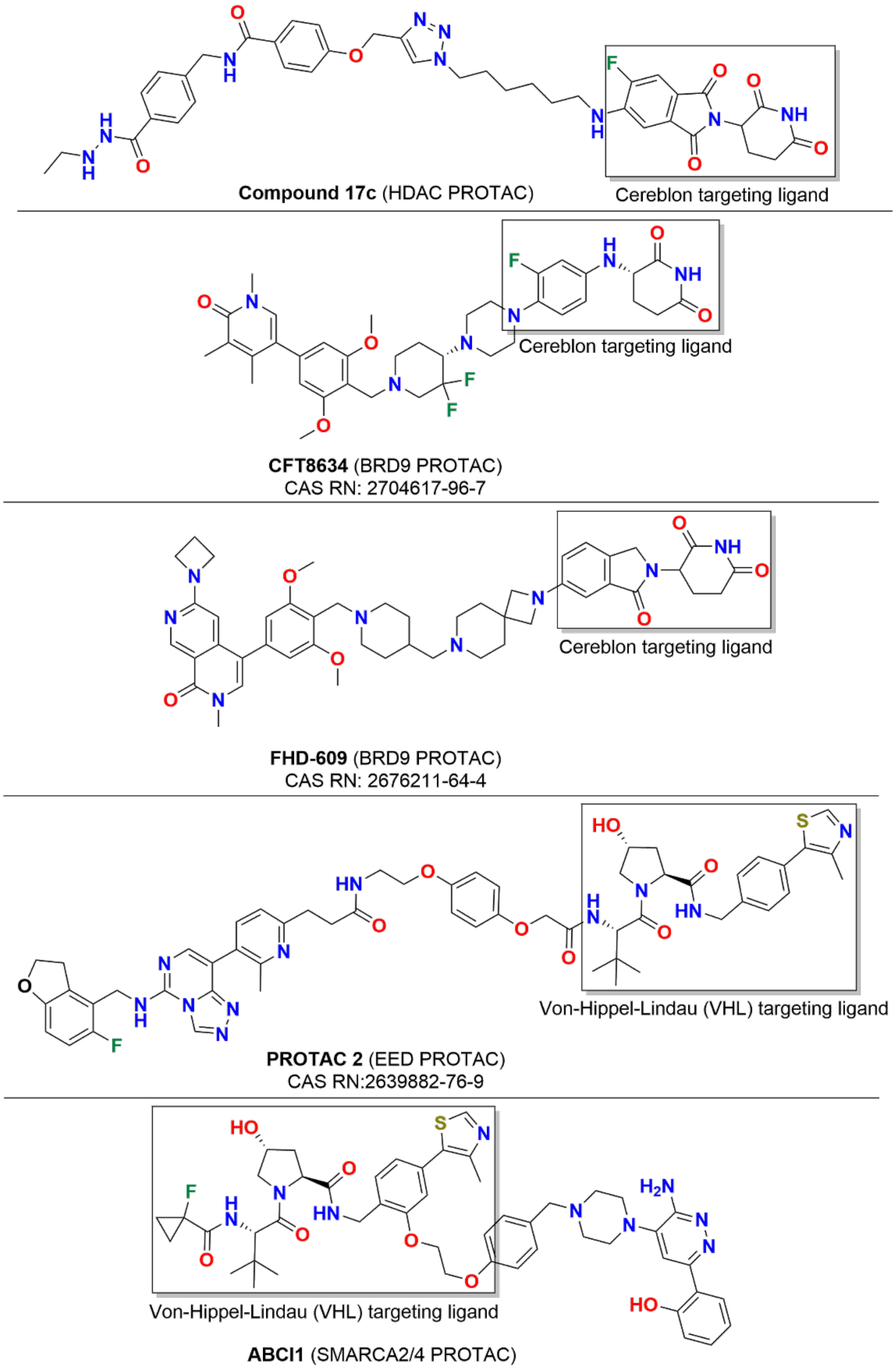

Figure S5. Structures of a few epigenetic PROTACs.

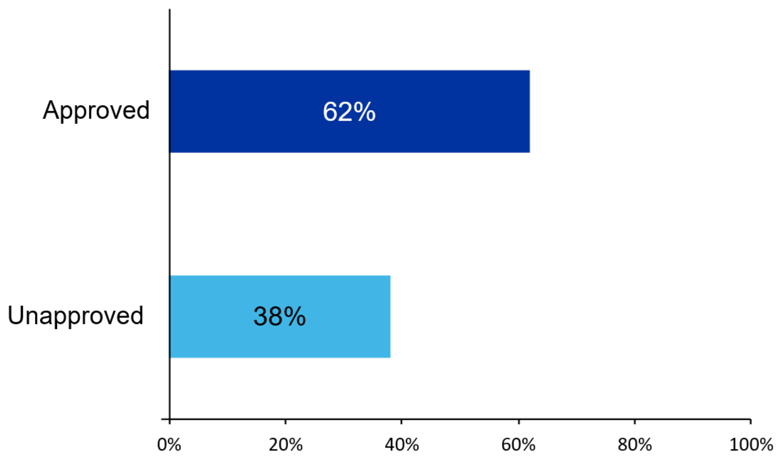

Figure S6. Overall percentage of therapeutic epigenetic drug clinical trials for both approved vs unapproved drugs.

Notable recent patents related to epigenetics

Summarized in **Table S1** are notable epigenetics-related recent patents published mostly in the recent decade. These examples were selected by CAS SMEs to highlight the range of discussed materials, compositions, methods, and technologies related to the field.

Table S1. Notable recent epigenetics-related patent application publications extracted from the CAS Content Collection.

Patent number | Publication year	Patent assignee	Title	Key features
US20250043329 | 2025	Columbia University	Methods of high throughput epigenomic mapping of normal and malignant hematopoietic stem and progenitor cells	The patent relates to methods of epigenomic mapping. Provided are reproducible, high throughput methods for determining chromatin structure and chromatin modifications in small sample volumes by use of focused bursts of ultrasonic acoustic energy for chromatin shearing before immunoprecipitation.[1]
WO2025049303 | 2025	Chroma Bio	Compositions and methods for epigenetic regulation	Disclosed is a nucleic acid, and strategies and methods of using such nucleic acid for regulating expression of the epigenetic editors and endogenous genes.[2]
WO2025038733 | 2025	Johns Hopkins University	Genome-wide repeat landscapes in cancer and cell-free DNA	Provided is a genome-wide approach for analyzing repeat landscapes in next generation sequencing. The approach termed ARTEMIS can assess over thousands of individual repeat types that occur genome-wide and span a plurality of subfamilies comprising a plurality of families. It is used to show that tumor-specific changes in repeats reflect a combination of structural and epigenetic changes in the cancer genome.[3]
WO2024238689 | 2024	Chroma Medicine, Inc.	Compositions and methods for multiplex epigenetic regulation	This patent provides epigenetic editors for repressing expression of two or more human genes, including in host cells and organisms. By altering expression of target genes, the disclosed editors may be used to generate allogeneic cells (e.g., T cells, NK cells, etc.) with reduced alloreactivity.[4]
WO2024178273 | 2024	University of California	Methods for epigenetic analysis	The patent discloses methods for conducting epigenomic profiling or chromatin mapping assays by blocking the antibodies used for isolating target cells or target nuclei. The methods further comprise isolating the target cells and/or target nuclei from a sample with an antibody-based assay.[5]

WO2024168114 | 2024	Battelle Memorial Institute	Technologies for individualized metagenomic profiling	The disclosed technologies include receiving a genome sequence for an individual, mapping it to generate a genome map compared to chimeric sequences associated with an identified pathogen, further generating a biomedical fingerprint by integrating the genome map, the chimera map, and the transposon map. The technologies may include mapping an epigenetic profile to the genome sequence to generate an epigenetic map.[6]
WO2024112806 | 2024	Moonwalk Biosciences	Generation and use of epigenetic maps for drug discovery	Provided are methods of generating a differential cellular state map for epigenetic editing. These methods utilize epigenetic maps of cells of different cellular states and cell types to identify unique methylation markers and patterns that may be contributors to a desired cellular state.[7]
CN119092140 | 2024	Beijing Hospital	Method for assessing male biological age and predicting aging based on DNA methylation data of peripheral blood by screening and establishing an epigenetic clock model	The aim of the invention is to provide a method for biological age assessment and aging prediction based on peripheral blood DNA methylation data, including screening of DNA methylation sites, acquisition of a modeling database, construction of epigenetic clock model, comparison and evaluation of the model, and further age assessment and aging prediction.[8]
WO2024064910 | 2024	Chroma Medicine	Compositions and methods for epigenetic regulation of HBV gene expression	This invention provides epigenetic editors, and strategies and methods of using such editors, for regulating expression of hepatitis B virus (HBV) genes, including HBV in host cells and organisms. The provided compositions and methods are useful to suppress viral function in infected cells.[9]
WO2023059922 | 2023	Micronoma, Inc.	Metaepigenomics-based disease diagnostics	The patent provides methods to identify disease-associated metaepigenomic biomarkers and methods to use them to accurately diagnose diseases from a tissue or liquid biopsy sample, including enriching a nucleic acid of that sample by affinity targeting, and sequencing the enriched nucleic acid.[10]
WO2023247789 | 2023	European Molecular Biology Laboratory	Complex and CRISPR-based modular tool for specific introduction of epigenetic modifications at target loci	The invention relates to a complex comprising a catalytically inactive site-specific nuclease linked to an array of effector domains with a specific chromatin modifying activity, wherein the effector domains are separated by a linker providing sufficient distance between the domains and the nuclease in order not to substantially interfere with their specific chromatin modifying activities, and the binding of the site-specific nuclease, as well as

			respective methods involving the complex and use of the complex.[11]
WO2022192890 | 2022	University of Pennsylvania	Preparation of DNA library for genome-wide analysis of protein-DNA interactions for personal epigenomics	The disclosure is aimed to detecting chromatin-protein interactions that regulate gene expression, for identifying protein-centric chromatin interactions from a small starting cell population. It provides methods for generating a sequencing library from a sample comprising a plurality of mammalian cells.[12]
WO2022272120 | 2022	University of California	Epigenetic clocks based on CpG methylation profiles of mammalian genomic DNA	In this patent DNA methylation profiles are used to develop biomarkers of aging known as epigenetic clocks, to accurately predicting chronological age as well as assessing other physiological factors associated with aging.[13]
CN111524603 | 2020	Wuxi Yana Health Management Consulting	Cancer risk detection method based on nutritional epigenomics and cancer prevention formula	The invention provides a cancer risk assessment method based on nutritional epigenomics, including performing correlation analysis on epigenetic clocks of cancer patients and distribution of anti-cancer components.[14]
WO2018007525 | 2018	Siemens Healthcare GmbH	Epigenome-wide association study identifies cardiac developmental gene patterning and a novel class of biomarkers for heart failure	The invention examines epigenome-wide cardiac and blood DNA methylation in conjunction with mRNA and whole-genome sequencing in patients with systolic heart failure due to dilated cardiomyopathy. It provides a large dataset of cardiac and blood DNA methylation profiles and identified key epigenomic patterns that are distinct fingerprints of human heart failure.[15]
IN201731039952 | 2018	Bose Institute	Systems level methods for epigenetic drug development for human diseases	The objective of the invention is to provide a novel system to identify drugs as per disease specific microRNAs, KEGG pathways, functional categories and disease specific targets, as well as to provide cost effective hardware system for identification and repositioning of drugs specific to human disease, with Alzheimer's disease as a workable example.[16]
WO2018132518 | 2018	Juno Therapeutics	Epigenetic analysis of cell therapy and related methods	Provided are methods of identifying genomic regions predictive of an outcome of treatment with a cell therapy and/or of a phenotype of function of the cells.[17]
WO2016123472 | 2016	Massachusetts Institute of Technology (MIT)	Methods for analyzing epigenomic characteristics	The patent provides techniques for analyzing characteristics associated with genomic regions, including epigenomic characteristics associated with genomic regions of an organism genome. Epigenomic data provide information about the dynamics of chromatin states in gene regulation, which can be used to identify putative targets to control cell- specific functions, and/or

			provide a novel approach to therapy for a disease or disorder, e.g., cancer.[18]
WO2015035112 | 2015	Johns Hopkins University	Cancer therapy via a combination of epigenetic modulation and immune modulation	Disclosed are cancer therapies combining epigenetic modulating agents with immune modulating agents, identified to provide an improved treatment regimen over single agent therapy. In particular, improved treatment of NSCLC in patients via administration of immune modulating agents anti-PD-1 antibody or anti-PD-L1 antibody, observed to show enhanced activity in combination with the epigenetic modulating agent 5-deoxyazacytidine.[19]

Table S2. Exemplary epi-drugs approved for clinical use

Drug (Trade name)	Approval year	CAS RN	Number of publications	PAT/JRN	Structural formula	Disease indications
			**DNMT inhibitors**			
Azacitidine (Vidaza)	2004	320-67-2	14,627	0.26	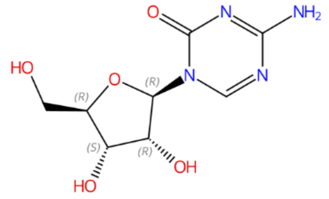	Cancer (refractory anemia (RA) or refractory anemia with ringed sideroblasts (RARS); refractory anemia with excess blasts (RAEB), refractory anemia with excess blasts in transformation (RAEB-T), and chronic myelomonocytic leukemia (CMMoL)) (ref)
Decitabine (Dacogen)	2006	2353-33-5	9168	0.31	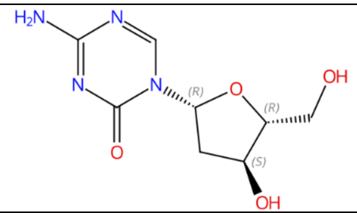	Cancer (myelodysplastic syndromes (MDS)) (ref)
			**HDAC inhibitors**			
Vorinostat (Zolinza)	2006	149647-78-9	7104	0.42	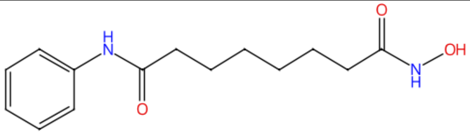	Cancer (cutaneous Tcell lymphoma (CTCL)) (ref)

Romidepsin (Istodax)	2009	128517-07-7	2425	0.77	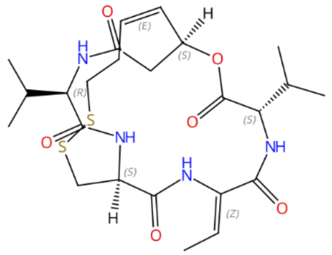	Cancer (cutaneous T-cell lymphoma (CTCL)) (ref)
Belinostat (Beleodaq)	2014	866323-14-0	1349	1.08	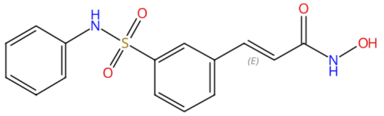	Cancer (relapsed or refractory peripheral T-cell lymphoma (PTCL)) (ref)
Panobinostat (Farydak) *withdrawn by FDA in 2022	2015	404950-80-7	2633	0.59	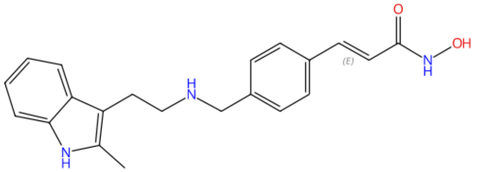	Cancer (multiple myeloma) (ref)
Givinostat (Duvyzat)	2024	497833-27-9	387	1.59	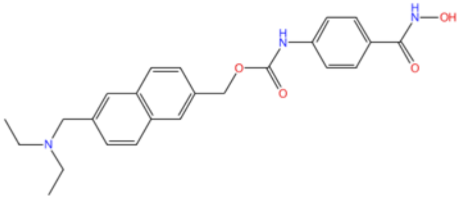	Duchenne muscular dystrophy (DMD) (ref)
Chidamide (Tucidinostat) *only approved by NMPA in China and Japan	2014 (China) (ref)<br><br>2021 (Japan) (ref)	1616493-44-7	642	0.60	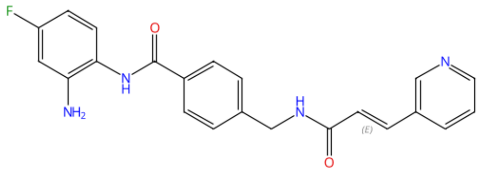	Cancer (2014: relapsed or refractory peripheral T cell lymphoma (PTCL) in China (ref), 2019: breast cancer in China (ref, ref), 2021: relapsed or refractory (R/R) T-cell leukemia (ATL) in Japan (ref), 2024: combination with R-CHOP for BCL2-positive diffuse large B-

						cell lymphoma (DLBCL) in China (ref))
**IDH inhibitors**						
Enasidenib (Idhifa)	2017	1446502-11-9	392	0.62	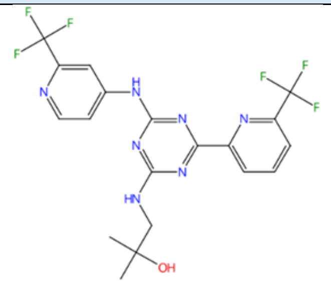	Cancer (relapsed or refractory acute myeloid leukemia (AML)) (ref)
Ivosidenib (Tibsovo)	2018	1448347-49-6	417	0.57	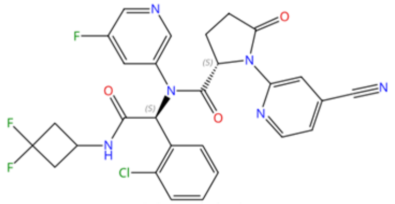	Cancer (AML, locally advanced or metastatic cholangiocarcinoma) (ref)
Olutasidenib (Rezlidhia)	2022	1887014-12-1	54	0.86	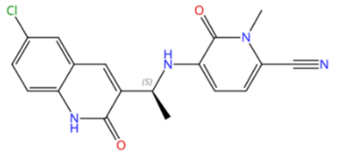	Cancer (relapsed or refractory AML) (ref)
Vorasidenib (Voranigo)	2024	1644545-52-7	90	0.29	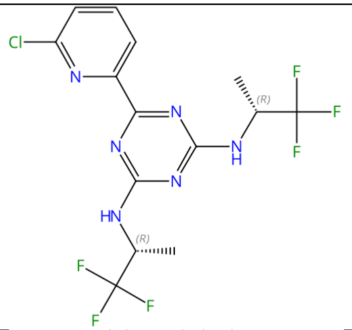	Cancer (astrocytoma or oligodendroglioma) (ref)

**EZH2 inhibitors**						
Tazemetostat (Tazverik)	2020	1403254-99-8	577	0.75	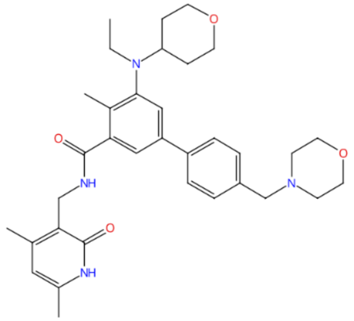	Cancer (metastatic or locally advanced epithelioid sarcoma, relapsed or refractory follicular lymphoma) (ref)
Valemetostat tosilate (Ezharmia) *only approved by PMDA in Japan	2022	1809336-39-7	63	0.97	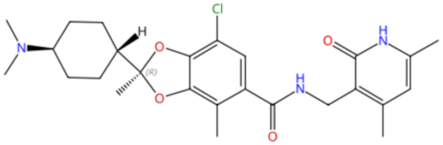	Cancer (non-Hodgkin lymphomas (NHL)) (ref)
**DRD inhibitor/ClpP activator**						
Dordaviprone (Modeyso)	2025	1616632-77-9	145	0.44	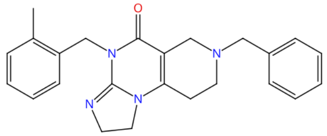	Vancer (diffuse midline glioma) (ref)
**Menin inhibitor**						
Revumenib (Revuforj)	2024	2169919-21-3	65	0.38	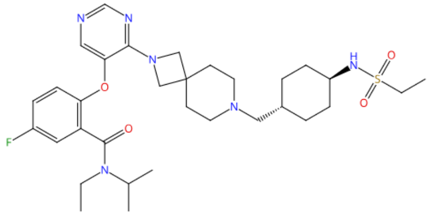	Cancer (relapsed or refractory acute leukemia) (ref)

ClpP, caseinolytic protease P; DNMT, DNA methyltransferase; DRD, Dopamine receptor; HDAC, histone deacetylase; EZH2, enhancer of zeste homolog 2; IDH, isocitrate dehydrogenase.

Table S3. Exemplary non-regulatory approved epigenetic drugs in the clinical trial pipeline highlighted by drug target. Drug indications, identifiers, and sponsoring organization with location are also included. This table contains highlighted agents and clinical trials currently active in the development pipeline, it is not exhaustive.

Drug Name	CAS RN	CT Phase	Indication	Identifier	Sponsoring Company
**DNMT inhibitors**					
Guadecitabine (SGI-110)	929901-49-5	Phase I	Cancer (Extrahepatic Bile Duct Adenocarcinoma, recurrent HCC, Pancreatic Cancer)	NCT03257761	University of Southern California
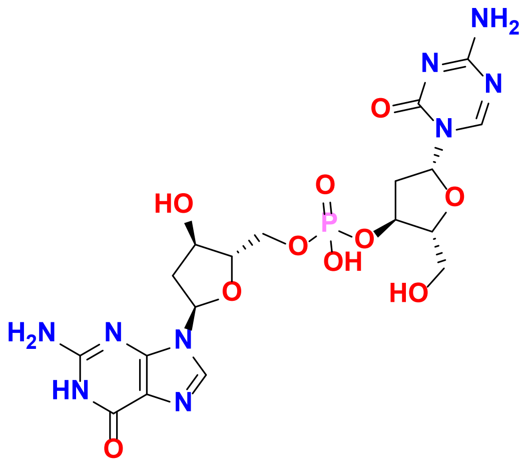		Phase I	Cancer (Lung)	NCT03220477	Memorial Sloan Kettering Cancer Center
		Phase I	Cancer (NSCLC)	NCT02998567	Royal Marsden NHS Foundation Trust
		Phase II	Cancer (Urothelial carcinoma)	NCT03179943	Fox Chase Cancer Center
		Phase II	Cancer (Locally Advanced Unresectable Primary Central Chondrosarcoma, Metastatic Primary Central Chondrosarcoma, Unresectable Primary Central Chondrosarcoma)	NCT04340843	National Cancer Institute (NCI)
		Phase I/II	Cancer (advanced kidney cancer, CCRCC)	NCT03308396	Big Ten Cancer Research Consortium

		Phase I/II	Cancer (CMML, MDS, AML)	NCT02935361	University of Southern California
**HDAC inhibitors**					
**KMT inhibitors**					
CPI-0209	2567686-02-4	Phase I	Cancer (platinum sensitive, recurrent ovarian cancer)	NCT05942300	University of Pittsburgh
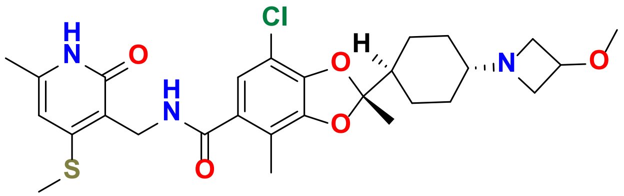		Phase I	Advanced (stage IB-IVB) mycosis fungoides (MF)/Sézary syndrome (SS)	NCT05944562	Washington University School of Medicine
		Phase I/II	Cancer (advanced solid tumors and lymphomas)	NCT04104776	Novartis Pharmaceuticals
PF-06821497 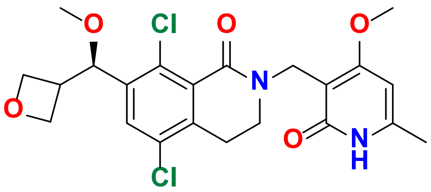	1844849-10-0	Phase I	Cancer (relapsed/refractory SCLC, castration resistant prostate cancer, and follicular lymphoma)	NCT03460977	Pfizer
XNW5004	NA	Phase I/II	Cancer (advanced solid tumors)	NCT06022757	Evopoint Biosciences Inc.
AXT-1003	NA	Phase I	Cancer (advanced malignancies)	NCT06484985	Axter Therapeutics (Beijing) Co., Ltd
KTX-1001	2604513-16-6	Phase I	Cancer (relapsed or refractory multiple myeloma)	NCT05651932	K36 Therapeutics, Inc.

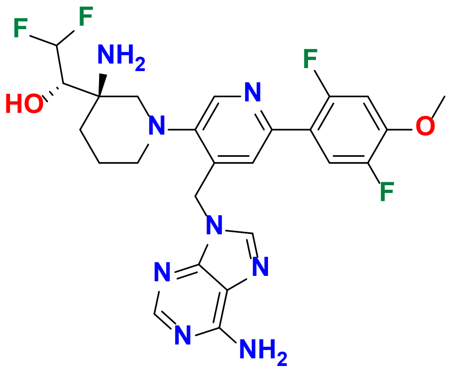					
**PRMT inhibitors**					
CTS-2190	NA	Phase I/II	Cancer (advanced or metastatic solid tumors)	NCT06224387	CytosinLab Therapeutics Co., Ltd.
TNG908 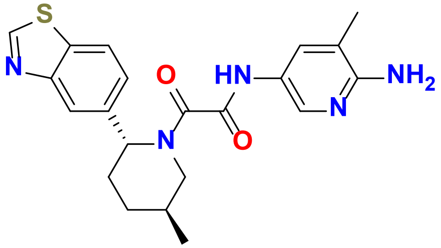	2760481-53-4	Phase I/II	Cancer (advanced or metastatic solid tumors known to have an advanced methylthioadenosine phosphorylase (MTAP) deletion)	NCT05275478	Tango Therapeutics, Inc.
MRTX1719 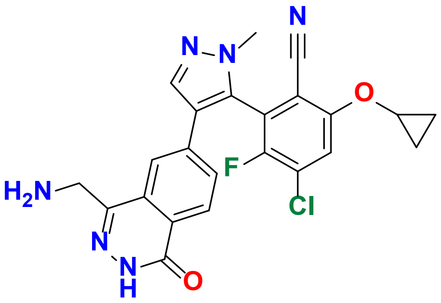	2629314-96-9	Phase I/II	Advanced, unresectable or metastatic solid tumor malignancy with homozygous deletion of the MTAP gene	NCT05245500	Bristol-Myers Squibb
AMG193	2962791-29-1	Phase I	Cancer (metastatic or locally advanced MTAP-deleted gastrointestinal,	NCT06360354	Amgen

			biliary tract, or pancreatic cancers)		
		Phase I	Cancer (metastatic or locally advanced MTAP-deleted thoracic tumors)	NCT06333951	Amgen
		Phase I/II	Cancer (metastatic or locally advanced MTAP-null solid tumors)	NCT05094336	Amgen
		Phase I/II	Cancer (metastatic or locally advanced MTAP-null solid tumors)	NCT05975073	Amgen
TNG462 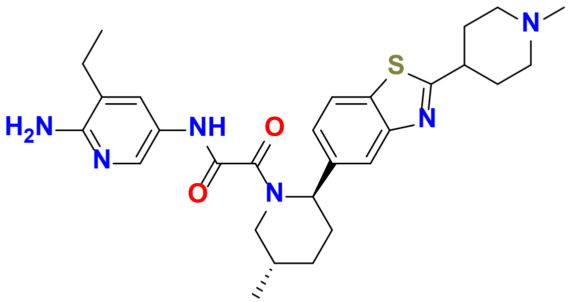	2760483-96-1	Phase I/II	Cancer (MTAP-null solid tumors)	NCT05732831	Tango Therapeutics, Inc.
SCR6920	NA	Phase I	Cancer (solid tumors, NHL)	NCT05528055	Jiangsu Simcere Pharmaceutical Co., Ltd.
SYHX-2001	NA	Phase I	Cancer (solid tumors)	NCT05407909	CSPC ZhongQi Pharmaceutical Technology Co., Ltd.
**KDM inhibitors**					
LH-1802	NA	Phase I	Cancer (AML, MDS)	CTR20222026	Jiangsu Lianhuan Pharmaceutical Co., Ltd.
**Chromatin remodellers**					
**SMARCA4/2 allosteric inhibitor**					

FHD-286 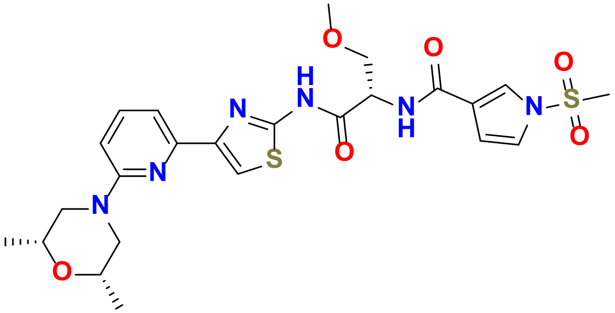	2671128-05-3	Phase I	Cancer (advanced hematologic malignancy, refractory AML, refractory CMML, refractory MDS, relapsed AML, relapsed CMML, relapsed MDS)	NCT04891757	Foghorn Therapeutics Inc.
**PROTAC**					
RNK-05047 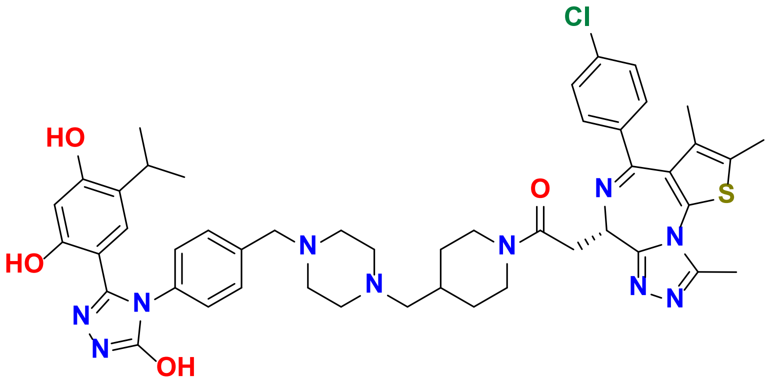	2503035-53-6	Phase I/II	Cancer (advanced solid tumor, DLBCL)	NCT05487170	Ranok Therapeutics (Hangzhou) Co., Ltd.
**CRISPR-based**					
Tune-401	NA	Phase I	Hepatitis B	NCT06671093	Tune Therapeutics, Inc.
**Combination**					
Azacitidine with or without lenalidomide or vorinostat	320-67-2, 191732-72-6, 149647-78-9	Phase II	Cancer (CMML, MDS, AML)	NCT01522976	National Cancer Institute (NCI)
Vorinostat, azacitidine	149647-78-9, 320-67-2	Phase I/II	Cancer (AML, CMML, MDS), refractory anemia)	NCT00392353	National Cancer Institute (NCI)
Durvalumab, pralatrexate, romidepsin and 5-azacitidine	1428935-60-7, 146464-95-1, 128517-07-7,	Phase I/II	Cancer (T-cell lymphoma)	NCT03161223	University of Virginia

	320-67-2				
5-Azacitidine, etinostat (MS-275)	320-67-2, 209783-80-2	Phase II	Cancer (AML)	NCT01305499	Case Comprehensive Cancer Center
Pembrolizumab, guadecitabine and mocetinostat	1374853-91-4, 929901-49-5, 726169-73-9	Phase I	Cancer (lung)	NCT03220477	Memorial Sloan Kettering Cancer Center

AML, acute myeloid leukemia; CMML, chronic myelomonocytic leukemia; DLBCL, diffuse large B-cell lymphoma; MDS, myelodysplastic syndrome; MF, mycosis fungoides; MTAP, methylthioadenosine phosphorylase; NHL, non-Hodgkin lymphoma; SCLC, small cell lung cancer; SS, Sézary syndrome;

Table S4. Summary of emerging technologies in epigenetics research.

Technology	Key features	Application
Single cell epigenomics[20-26]	Allows analysis of epigenetic modifications at the resolution of individual cells, revealing cell-to-cell variability.<br>Techniques include:<br>- Single-cell ATAC-seq (scATAC-seq):[27-29] profiles chromatin accessibility in single cells, revealing cell-type-specific regulatory elements.<br>- Single-cell ChIP-seq (scChIP-seq):[29] maps histone modifications or transcription factor binding in individual cells.<br>- Single-cell DNA methylation sequencing:[30] measures DNA methylation patterns at single-cell resolution.	- Understanding heterogeneity in cancer, immune responses, and brain development.<br>- Mapping epigenetic landscapes in rare cell populations, such as stem cells or circulating tumor cells.
Multi-omics integration[31, 32]	Combines epigenomic data with transcriptomics, proteomics, and metabolomics for a holistic view of cellular function.<br>Techniques include:<br>- CUT&RUN (cleavage under targets and release using nuclease):[33] profiles histone modifications and chromatin-binding proteins.<br>- Hi-C[34] and ChIP-seq integration: links chromatin architecture with transcription factor binding.	- Deciphering how epigenetic modifications interact with other molecular layers to regulate gene expression.<br>- Identifying biomarkers and pathways in complex diseases.
Epigenome editing tools[35, 36]	Precision tools allow targeted manipulation of epigenetic marks without altering DNA sequences.<br>Technologies include:<br>- CRISPR/dCas9-based systems: fuse dCas9 (dead Cas9) with epigenetic effectors (e.g., DNMTs, TETs, HDACs) to add or remove specific modifications.[37, 38]<br>- TALE[39] and zinc-finger proteins:[36, 40] provide alternative targeting platforms for modifying specific loci.	- Investigating causal roles of specific epigenetic modifications.<br>- Developing epigenetic therapies for diseases like cancer, neurodegenerative disorders, and autoimmune conditions.
Advanced imaging and visualization techniques[41, 42]	High-resolution imaging tools enable direct observation of epigenetic modifications and chromatin dynamics in real time.<br>Techniques include:<br>- Super-resolution microscopy: techniques like STORM[43] and PALM[44] to visualize epigenetic modifications at the nanoscale.<br>- Live-cell imaging with fluorescent epigenetic probes: tracks changes in histone modifications or DNA methylation in living cells.	- Studying chromatin remodeling during cellular processes like differentiation, replication, and stress responses.<br>- Understanding spatial organization of epigenetic marks in the nucleus.

Long-read sequencing technologies[45, 46]	Technologies like PacBio and Oxford Nanopore allow direct detection of DNA and RNA modifications without chemical conversion.<br>Offers greater accuracy for repetitive regions and epigenetic marks missed by short-read sequencing.	- Comprehensive mapping of epigenetic modifications, including m6A and m5C, across genomes and transcriptomes.<br>- Investigating epigenetic changes in complex regions like centromeres and telomeres.
Artificial intelligence (AI) and machine learning[47-50]	AI and machine learning are used to analyze and interpret large-scale epigenomic datasets.<br>They are capable of identifying patterns, predicting functional effects of modifications, and integrating multi-omics data.	- Predicting disease-associated epigenetic changes from genomic data.<br>- Accelerating drug discovery by identifying epigenetic drug targets.
Epigenetic biomarker discovery platforms[51-54]	High-throughput technologies for identifying and validating epigenetic biomarkers in diseases.<br>Includes liquid biopsy methods for detecting circulating methylated DNA or histone modifications in bodily fluids.	- Early diagnosis and prognosis of cancers, cardiovascular diseases, and neurodegenerative disorders.<br>- Monitoring therapeutic responses in real time.
Synthetic biology for epigenetics[55-58]	Synthetic constructs mimic or modulate epigenetic regulatory networks.<br>Includes synthetic transcription factors and artificial chromatin remodelers.	- Reprogramming cell fate for regenerative medicine.<br>- Engineering epigenetic switches for research and therapeutic use.
High-throughput functional epigenomics[59-62]	Systems like Perturb-seq and CRISPR screens link epigenetic modifications to functional outcomes.[63] (ref)<br>Combines genetic and epigenetic perturbations with transcriptomic analysis.	- Identifying genes and pathways regulated by specific epigenetic modifications.<br>- Discovering novel drug targets for epigenetic therapies.
Organoid[64, 65] and *in vivo* epigenetics models[66-68]	Organoids and animal models with engineered epigenomes provide physiologically relevant systems to study epigenetic mechanisms.<br>Technologies like epigenome-edited mice enable exploration of developmental and disease-specific epigenetics.	- Modeling epigenetic dysregulation in diseases like cancer, Alzheimer's, and diabetes.<br>- Testing epigenetic therapies in preclinical settings.

References

(1) Viny, A. D. Methods of High Throughput Epigenomic Mapping of Normal and Malignant Hematopoietic Stem and Progenitor Cells. United States US20250043329, 2025.
(2) Reddy, N. C.; Myer, V. Compositions and Methods for Epigenetic Regulation. United States WO2025049303, 2025.
(3) Velculescu, V. E.; Annapragada, A.; Scharpf, R. B. Genome-wide Repeat Landscapes in Cancer and Cell-free DNA. United States WO2025038733, 2025.
(4) Myer, V.; Schafer, J. L.; Jaffe, A. B.; Maeder, M.; Friedland, A. Compositions and Methods for Multiplex Epigenetic Regulation. United States WO2024238689, 2024.
(5) Hamid, S.; Lim, D. Methods for Epigenetic Analysis. United States WO2024178273, 2024.
(6) Howland, C.; Bartling, C. M.; Gemler, B.; Fullerton, P.; Schuetter, J.; Mukherjee, S.; Spurbeck, R. R. Technologies for Individualized Metagenomic Profiling. United States WO2024168114, 2024.
(7) Jamshidi, A.; Valley, J. K.; Kishton, R. J.; Gross, S.; Mendez Romero, P. J. Generation and Use of Epigenetic Maps for Drug Discovery. United States WO2024112806, 2024.
(8) Sun, L.; Zhou, Z.; Lv, P.; Yang, R.; Liu, H.; Chen, G.; Yu, B.; Hou, Y.; Zhou, Q.; Zhu, X.; et al. Method for Assessing Male Biological Age and Predicting Aging Based on DNA Methylation Data of Peripheral Blood by Screening and Establishing an Epigenetic Clock Model. China CN119092140, 2024.
(9) Jaffe, A. B.; Abubucker, N.; Anglero-Rodriguez, Y.; Myer, V.; Lombardo, A. L.; Cappelluti, M. A. Compositions and Methods for Epigenetic Regulation of HBV Gene Expression. United States WO2024064910, 2024.
(10) Adams, E.; Wandro, S.; Fraraccio, S.; Singh-Taylor, A. Metaepigenomics-based Disease Diagnostics. United States WO2023059922, 2023.
(11) Hackett, J.; Policarpi, C. Complex and CRISPR-based Modular Tool for Specific Introduction of Epigenetic Modifications at Target Loci. Germany WO2023247789, 2023.
(12) Leung, T. H.-C.; Sati, S.; Kim, H. Preparation of DNA Library for Genome-wide Analysis of Protein-DNA Interactions for Personal Epigenomics. United States WO2022192890, 2022.
(13) Horvath, S. Epigenetic Clocks Based on CpG Methylation Profiles of Mammalian Genomic DNA. United States WO2022272120, 2022.
(14) Zeng, J. Cancer Risk-detection Method Based on Nutritional Epigenomics and Cancer-prevention Formula. China CN111524603, 2020.
(15) Posch, A. E.; Meder, B.; Haas, J.; Katus, H. A.; Wuerstle, M.; Sedaghat-Hamedani, F. Epigenome-wide Association Study Identifies Cardiac Developmental Gene Patterning and a Novel Class of Biomarkers for Heart Failure. Germany WO2018007525, 2018.
(16) Roy, D.; Basu, A. Systems Level Methods for Epigenetic Drug Development for Human Diseases. India IN201731039952, 2018.
(17) Bonyhadi, M. L.; Kugler, D. G.; Johnstone, T. G.; Hause, R. J. J. Epigenetic Analysis of Cell Therapy and Related Methods. United States WO2018132518, 2018.
(18) Meuleman, W. Methods for Analyzing Epigenomic Characteristics. United States WO2016123472, 2016.
(19) Baylin, S. B.; Pardoll, D. M.; Topalian, S. L. Cancer Therapy Via a Combination of Epigenetic Modulation and Immune Modulation. United States WO2015035112, 2015.
(20) Hu, Y.; Shen, F.; Yang, X.; Han, T.; Long, Z.; Wen, J.; Huang, J.; Shen, J.; Guo, Q. Single-cell Sequencing Technology Applied to Epigenetics for the Study of Tumor Heterogeneity. *Clinical Epigenetics* **2023**, *15* (1), 161. DOI: 10.1186/s13148-023-01574-x.
(21) Mazan-Mamczarz, K.; Ha, J.; De, S.; Sen, P. Single-Cell Analysis of the Transcriptome and Epigenome. *Methods in Molecular Biology* **2022**, *2399*, 21-60. DOI: 10.1007/978-1-0716-1831-8_3.
(22) Clark, S. J.; Lee, H. J.; Smallwood, S. A.; Kelsey, G.; Reik, W. Single-Cell Epigenomics: Powerful New Methods for Understanding Gene Regulation and Cell Identity. *Genome Biology* **2016**, *17*, 72. DOI: 10.1186/s13059-016-0944-x.
(23) Kelsey, G.; Stegle, O.; Reik, W. Single-cell Epigenomics: Recording the Past and Predicting the Future. *Science* **2017**, *358* (6359), 69-75. DOI: 10.1126/science.aan6826.
(24) Zhang, D.; Deng, Y.; Kukanja, P.; Agirre, E.; Bartosovic, M.; Dong, M.; Ma, C.; Ma, S.; Su, G.; Bao, S.; et al. Spatial Epigenome-transcriptome Co-profiling of Mammalian Tissues. *Nature* **2023**, *616* (7955), 113-122. DOI: 10.1038/s41586-023-05795-1.
(25) Preissl, S.; Gaulton, K. J.; Ren, B. Characterizing Cis-regulatory Elements Using Single-cell Epigenomics. *Nature Reviews Genetics* **2023**, *24* (1), 21-43. DOI: 10.1038/s41576-022-00509-1.

(26) Schwartzman, O.; Tanay, A. Single-cell Epigenomics: Techniques and Emerging Applications. *Nature Reviews Genetics* **2015**, *16* (12), 716-726. DOI: 10.1038/nrg3980.
(27) Ay-Berthomieu, A.-S. *Beginner's Guide to Understanding Single-Cell ATAC-Seq*. 2020. https://www.activemotif.com/blog-single-cell-atac-seq (accessed Feb 16, 2025).
(28) Tjoonk, N. *Single-Cell ATAC-seq: The Basics*. 2023. https://www.scdiscoveries.com/blog/knowledge/single-cell-atac-seq-the-basics/ (accessed Feb 16, 2025).
(29) Ma, S.; Zhang, Y. Profiling Chromatin Regulatory Landscape: Insights into the Development of ChIP-seq and ATAC-seq. *Molecular Biomedicine* **2020**, *1* (1), 9. DOI: 10.1186/s43556-020-00009-w.
(30) Karemaker, I. D.; Vermeulen, M. Single-Cell DNA Methylation Profiling: Technologies and Biological Applications. *Trends in Biotechnology* **2018**, *36* (9), 952-965. DOI: 10.1016/j.tibtech.2018.04.002.
(31) Chen, C.; Wang, J.; Pan, D.; Wang, X.; Xu, Y.; Yan, J.; Wang, L.; Yang, X.; Yang, M.; Liu, G. P. Applications of Multi-Omics Analysis in Human Diseases. *MedComm (2020)* **2023**, *4* (4), e315. DOI: 10.1002/mco2.315.
(32) Ruprecht, N. A.; Kennedy, J. D.; Bansal, B.; Singhal, S.; Sens, D.; Maggio, A.; Doe, V.; Hawkins, D.; Campbel, R.; O'Connell, K.; et al. Transcriptomics and Epigenetic Data Integration Learning Module on Google Cloud. *Briefings in Bioinformatics* **2024**, *25* (Supplement_1). DOI: 10.1093/bib/bbae352.
(33) Yu, F.; Sankaran, V. G.; Yuan, G. C. CUT&RUNTools 2.0: A Pipeline for Single-cell and Bulk-level CUT&RUN and CUT&Tag Data Analysis. *Bioinformatics* **2021**, *38* (1), 252-254. DOI: 10.1093/bioinformatics/btab507.
(34) Lajoie, B. R.; Dekker, J.; Kaplan, N. The Hitchhiker's Guide to Hi-C Analysis: Practical Guidelines. *Methods* **2015**, *72*, 65-75. DOI: 10.1016/j.ymeth.2014.10.031.
(35) McCutcheon, S. R.; Rohm, D.; Iglesias, N.; Gersbach, C. A. Epigenome Editing Technologies for Discovery and Medicine. *Nature Biotechnology* **2024**, *42* (8), 1199-1217. DOI: 10.1038/s41587-024-02320-1.
(36) Waryah, C. B.; Moses, C.; Arooj, M.; Blancafort, P. Zinc Fingers, TALEs, and CRISPR Systems: A Comparison of Tools for Epigenome Editing. *Methods in Molecular Biology* **2018**, *1767*, 19-63. DOI: 10.1007/978-1-4939-7774-1_2.
(37) Josipovic, G.; Tadic, V.; Klasic, M.; Zanki, V.; Beceheli, I.; Chung, F.; Ghantous, A.; Keser, T.; Madunic, J.; Boskovic, M.; et al. Antagonistic and Synergistic Epigenetic Modulation Using Orthologous CRISPR/dCas9-based Modular System. *Nucleic Acids Research* **2019**, *47* (18), 9637-9657. DOI: 10.1093/nar/gkz709.
(38) Policarpi, C.; Munafo, M.; Tsagkris, S.; Carlini, V.; Hackett, J. A. Systematic Epigenome Editing Captures the Context-dependent Instructive Function of Chromatin Modifications. *Nature Genetics* **2024**, *56* (6), 1168-1180. DOI: 10.1038/s41588-024-01706-w.
(39) Kubik, G.; Summerer, D. TALEored Epigenetics: A DNA-Binding Scaffold for Programmable Epigenome Editing and Analysis. *Chembiochem* **2016**, *17* (11), 975-980. DOI: 10.1002/cbic.201600072.
(40) Zhang, R.; Yao, T.; Fan, M.; Jiang, X.; Wang, K.; Cui, M.; Bing, K.; Xia, X. Precision Scalpels for the Epigenome: Next-gen Editing Tools in Targeted Therapies. *Frontiers in Medicine* **2025**, *12*, 1613722. DOI: 10.3389/fmed.2025.1613722.
(41) Thompson, A. C.; Wopereis, J. L. M.; Tekle, Y. I.; Katz, L. A. Visualizing Epigenetics: A Review of Microscopy Techniques for Investigating DNA Methylation Patterns, Chromatin Structure, and Gene Expression. *Microscopy and Microanalysis* **2025**, *31* (2). DOI: 10.1093/mam/ozaf017.
(42) Moshareva, M. A.; Lukyanov, K. A.; Putlyaeva, L. V. Fluorescence Imaging of Epigenetic Genome Modifications. *Biochemical and Biophysical Research Communications* **2022**, *622*, 86-92. DOI: 10.1016/j.bbrc.2022.07.014.
(43) Xu, J.; Ma, H.; Liu, Y. Stochastic Optical Reconstruction Microscopy (STORM). *Current Protocols in Cytometry* **2017**, *81*, 12 46 11-12 46 27. DOI: 10.1002/cpcy.23.
(44) Lippincott-Schwartz, J.; Manley, S.; Burnette, D.; Gillette, J.; Patterson, G. PALM-Based Super-Resolution Imaging and its Applications. *Biophysical Journal* **2010**, *98*, 619A. DOI: 10.1016/j.bpj.2009.12.3382.
(45) Lucas, M. C.; Novoa, E. M. Long-read Sequencing in the Era of Epigenomics and Epitranscriptomics. *Nature Methods* **2023**, *20* (1), 25-29. DOI: 10.1038/s41592-022-01724-8.
(46) Liu, T.; Conesa, A. Profiling the Epigenome Using Long-read Sequencing. *Nature Genetics* **2025**, *57* (1), 27-41. DOI: 10.1038/s41588-024-02038-5.
(47) Brasil, S.; Neves, C. J.; Rijoff, T.; Falcao, M.; Valadao, G.; Videira, P. A.; Dos Reis Ferreira, V. Artificial Intelligence in Epigenetic Studies: Shedding Light on Rare Diseases. *Frontiers in Molecular Biosciences* **2021**, *8*, 648012. DOI: 10.3389/fmolb.2021.648012.
(48) Arslan, E.; Schulz, J.; Rai, K. Machine Learning in Epigenomics: Insights into Cancer Biology and Medicine. *Biochimica et Biophysica Acta Reviews on Cancer* **2021**, *1876* (2), 188588. DOI: 10.1016/j.bbcan.2021.188588.
(49) Rauschert, S.; Raubenheimer, K.; Melton, P. E.; Huang, R. C. Machine Learning and Clinical Epigenetics: A Review of Challenges for Diagnosis and Classification. *Clinical Epigenetics* **2020**, *12* (1), 51. DOI: 10.1186/s13148-020-00842-4.

(50) Tahir, M.; Norouzi, M.; Khan, S. S.; Davie, J. R.; Yamanaka, S.; Ashraf, A. Artificial Intelligence and Deep Learning Algorithms for Epigenetic Sequence Analysis: A Review for Epigeneticists and AI Experts. *Computers in Biology and Medicine* **2024**, *183*, 109302. DOI: 10.1016/j.compbiomed.2024.109302.
(51) Garcia-Gimenez, J. L.; Seco-Cervera, M.; Tollefsbol, T. O.; Roma-Mateo, C.; Peiro-Chova, L.; Lapunzina, P.; Pallardo, F. V. Epigenetic Biomarkers: Current Strategies and Future Challenges for Their Use in the Clinical Laboratory. *Critical Reviews in Clinical Laboratory Sciences* **2017**, *54* (7-8), 529-550. DOI: 10.1080/10408363.2017.1410520.
(52) Dirks, R. A.; Stunnenberg, H. G.; Marks, H. Genome-Wide Epigenomic Profiling for Biomarker Discovery. *Clinical Epigenetics* **2016**, *8*, 122. DOI: 10.1186/s13148-016-0284-4.
(53) Skinner, M. K. Epigenetic Biomarkers for Disease Susceptibility and Preventative Medicine. *Cell Metabolism* **2024**, *36* (2), 263-277. DOI: 10.1016/j.cmet.2023.11.015.
(54) Gao, J.; Shi, W.; Wang, J.; Guan, C.; Dong, Q.; Sheng, J.; Zou, X.; Xu, Z.; Ge, Y.; Yang, C.; et al. Research Progress and Applications of Epigenetic Biomarkers in Cancer. *Frontiers in Pharmacology* **2024**, *15*, 1308309. DOI: 10.3389/fphar.2024.1308309.
(55) Franklin, K. A.; Haynes, K. A. Chapter 10 - Synthetic Biology and Cell Engineering—Deriving New Insights into Cancer Epigenetics. In *Epigenetic Cancer Therapy*, Second ed.; Gray, S. G. Ed.; Academic Press, 2023; pp 195-210.
(56) Komera, I.; Chen, X.; Liu, L.; Gao, C. Microbial Synthetic Epigenetic Tools Design and Applications. *ACS Synthetic Biology* **2024**, *13* (6), 1621-1632. DOI: 10.1021/acssynbio.4c00125.
(57) Maier, J. A. H.; Mohrle, R.; Jeltsch, A. Design of Synthetic Epigenetic Circuits Featuring Memory Effects and Reversible Switching Based on DNA Methylation. *Nature Communications* **2017**, *8*, 15336. DOI: 10.1038/ncomms15336.
(58) Rodriguez-Escamilla, Z.; Martinez-Nunez, M. A.; Merino, E. Epigenetics Knocks on Synthetic Biology's Door. *FEMS Microbiology Letters* **2016**, *363* (17). DOI: 10.1093/femsle/fnw191.
(59) Winkler, K. R.; Mizrahi, V.; Warner, D. F.; De Wet, T. J. High-throughput Functional Genomics: A (Myco)bacterial Perspective. *Molecular Microbiology* **2023**, *120* (2), 141-158. DOI: 10.1111/mmi.15103.
(60) R, E. Y.; J, P. G.; Dahmane, N. Developments in High-throughput Functional Epigenomics: CRISPR-Single-cell Assay for Transposase-accessible Chromatin Using Sequencing Screens. *Epigenomics* **2022**, *14* (11), 645-649. DOI: 10.2217/epi-2022-0093.
(61) Sheng, T.; Ho, S. W. T.; Ooi, W. F.; Xu, C.; Xing, M.; Padmanabhan, N.; Huang, K. K.; Ma, L.; Ray, M.; Guo, Y. A.; et al. Integrative Epigenomic and High-throughput Functional Enhancer Profiling Reveals Determinants of Enhancer Heterogeneity in Gastric Cancer. *Genome Medicine* **2021**, *13* (1), 158. DOI: 10.1186/s13073-021-00970-3.
(62) Bock, C. Toward High-Throughput Functional Epigenomics Using CRISPR Single-Cell Sequencing. *Experimental Hematology* **2017**, *53*. DOI: 10.1016/j.exphem.2017.06.035.
(63) Dixit, A.; Parnas, O.; Li, B.; Chen, J.; Fulco, C. P.; Jerby-Arnon, L.; Marjanovic, N. D.; Dionne, D.; Burks, T.; Raychowdhury, R.; et al. Perturb-Seq: Dissecting Molecular Circuits with Scalable Single-Cell RNA Profiling of Pooled Genetic Screens. *Cell* **2016**, *167* (7), 1853-1866 e1817. DOI: 10.1016/j.cell.2016.11.038.
(64) Zhao, Z.; Chen, X.; Dowbaj, A. M.; Sljukic, A.; Bratlie, K.; Lin, L.; Fong, E. L. S.; Balachander, G. M.; Chen, Z.; Soragni, A.; et al. Organoids. *Nature Reviews Methods Primers* **2022**, *2*. DOI: 10.1038/s43586-022-00174-y.
(65) Kim, J.; Koo, B. K.; Knoblich, J. A. Human Organoids: Model Systems for Human Biology and Medicine. *Nature Reviews Molecular Cell Biology* **2020**, *21* (10), 571-584. DOI: 10.1038/s41580-020-0259-3.
(66) Edgar, R. D.; Perrone, F.; Foster, A. R.; Payne, F.; Lewis, S.; Nayak, K. M.; Kraiczy, J.; Cenier, A.; Torrente, F.; Salvestrini, C.; et al. Culture-Associated DNA Methylation Changes Impact on Cellular Function of Human Intestinal Organoids. *Cellular and Molecular Gastroenterology and Hepatology* **2022**, *14* (6), 1295-1310. DOI: 10.1016/j.jcmgh.2022.08.008.
(67) Wang, Y. W.; Hu, N.; Li, X. H. Genetic and Epigenetic Regulation of Brain Organoids. *Frontiers in Cell and Developmental Biology* **2022**, *10*, 948818. DOI: 10.3389/fcell.2022.948818.
(68) Dennison, T. W.; Edgar, R. D.; Payne, F.; Nayak, K. M.; Ross, A. D. B.; Cenier, A.; Glemas, C.; Giachero, F.; Foster, A. R.; Harris, R.; et al. Patient-Derived Organoid Biobank Identifies Epigenetic Dysregulation of Intestinal Epithelial MHC-I as a Novel Mechanism in Severe Crohn's Disease. *Gut* **2024**, *73* (9), 1464-1477. DOI: 10.1136/gutjnl-2024-332043.
